# Development of a one-shot dual aptamer-based fluorescence nanosensor for rapid, sensitive, and label-free detection of periostin

**DOI:** 10.1038/s41598-023-37418-0

**Published:** 2023-06-23

**Authors:** Jonghoon Park, Changill Ban

**Affiliations:** Department of Chemistry, Pohang University of Science and Technology, 77, Cheongam-ro, Nam-gu, Pohang, Gyeongbuk 37673 Republic of Korea

**Keywords:** Biosensors, Nanoparticles

## Abstract

Periostin is associated with several diseases, including cancers. Therefore, monitoring blood periostin levels is a powerful tool for diagnosing various diseases and identifying their severity. However, conventional detection methods pose several challenges, including high costs. To address these issues, we developed a novel one-shot dual aptamer-based fluorescence nanosensor for detecting periostin. The proposed nanosensor facilitates rapid, label-free, and sensitive detection of periostin using gold nanoprobes constructed by rhodamine-b isothiocyanate, PL2_trunc_ aptamer, and gold nanoparticles and silver nanoprobes fabricated by the PL5_trunc_ aptamer and silver nanoparticles. The two nanoprobes form a core-satellite structure by interacting with periostin, and the nanosensor detects periostin through the fluorescence regenerated by the increased proximity between them. The nanosensor successfully detected periostin with remarkable detection limits of 106.68 pM in buffer and 463.3 pM in serum-spiked conditions within 30 min without additional washing or signal amplification processes. Considering serum periostin levels in various diseases, the proposed nanosensor provides a suitable method for identifying patients with various diseases and determining disease severity. Moreover, the platform can be helpful as a practical method for on-site medical diagnosis because it can be adapted to detect other biomarkers simply by replacing the aptamer with other detection probes.

## Introduction

Monitoring blood biomarkers is a powerful tool for the diagnosis and prognosis of diseases such as cancer and diabetes^1^. Blood is a unique pathological sample as it can be obtained without causing much pain to the patient and carries several forms of disease biomarkers, such as proteins, nucleic acids, tumor cells, and metabolites^2,3^. Among them, protein biomarkers are considered the most useful, as they are directly connected to cellular functions and can be easily targeted^3,4^. Periostin is an extracellular matrix protein involved in various pathophysiological processes, including cell proliferation and cancer pathogenesis^5,6^. The periostin level in the blood is elevated in patients with diseases such as non-small cell lung cancer, obstructive sleep apnea-hypopnea syndrome, and diabetic retinopathy^7–9^. Therefore, highly sensitive methods for detecting periostin in the blood are in demand for the diagnosis and prognosis of these diseases.

Conventional and widely used detection methods for protein biomarker include mass spectroscopy, surface-enhanced Raman scattering (SERS), microarray, and enzyme-linked immunosorbent assay (ELISA)^10–14^. These methods are immunoassay-based utilizing antibodies and enzymes and have high sensitivity and selectivity^4^. However, they have several limitations, including the need for expensive equipment and experts, complicated steps such as signal amplification and washing, and high costs^15^. Moreover, cost-effective and sensitive alternative detection systems for periostin have not yet been developed. Consequently, the development of new molecular probes and detection systems that can replace antibodies for economical, simple, and rapid detection is desired.

Nanotechnology presents an innovative way to overcome the limitations of conventional methods using small materials, including aptamers and metal nanoparticles. Aptamers are single-stranded oligonucleotides (ssDNA) that can specifically bind to a target molecule owing to their three-dimensional structures^16^. Aptamers can be developed from a randomized pool of libraries using the systematic evolutions of ligands by exponential enrichment (SELEX)^17^. Aptamers offer many advantages over antibodies, including large-scale synthesis, low cost, no batch-to-batch variation, and high chemical and thermal stability^18,19^. Owing to these advantages, aptamers are widely applied in developing on-site sensors, modularization, and convenient multifunctional biosensors^20^. Meanwhile, metal nanoparticles (NPs) can act as quenchers (~ 5 nm distance) or enhancers (10–90 nm distance) depending on their distance from the fluorophore due to their Förster resonance energy transfer (FRET) and metal-enhanced fluorescence (MEF) properties by a localized surface plasmon resonance (SPR) energy^21^. In addition, they have a high surface-to-volume ratio^22^. Moreover, metal NPs can act as a fluorescence regenerator in which the MEF effect of one NP can regenerate the quenched fluorescence of the fluorophore conjugated to another NP when both NPs form a core-satellite structure^23^. Therefore, metal NPs have been widely applied in various fields, including bioimaging, biosensors, and biomedical applications^24^.

**Figure 1:**
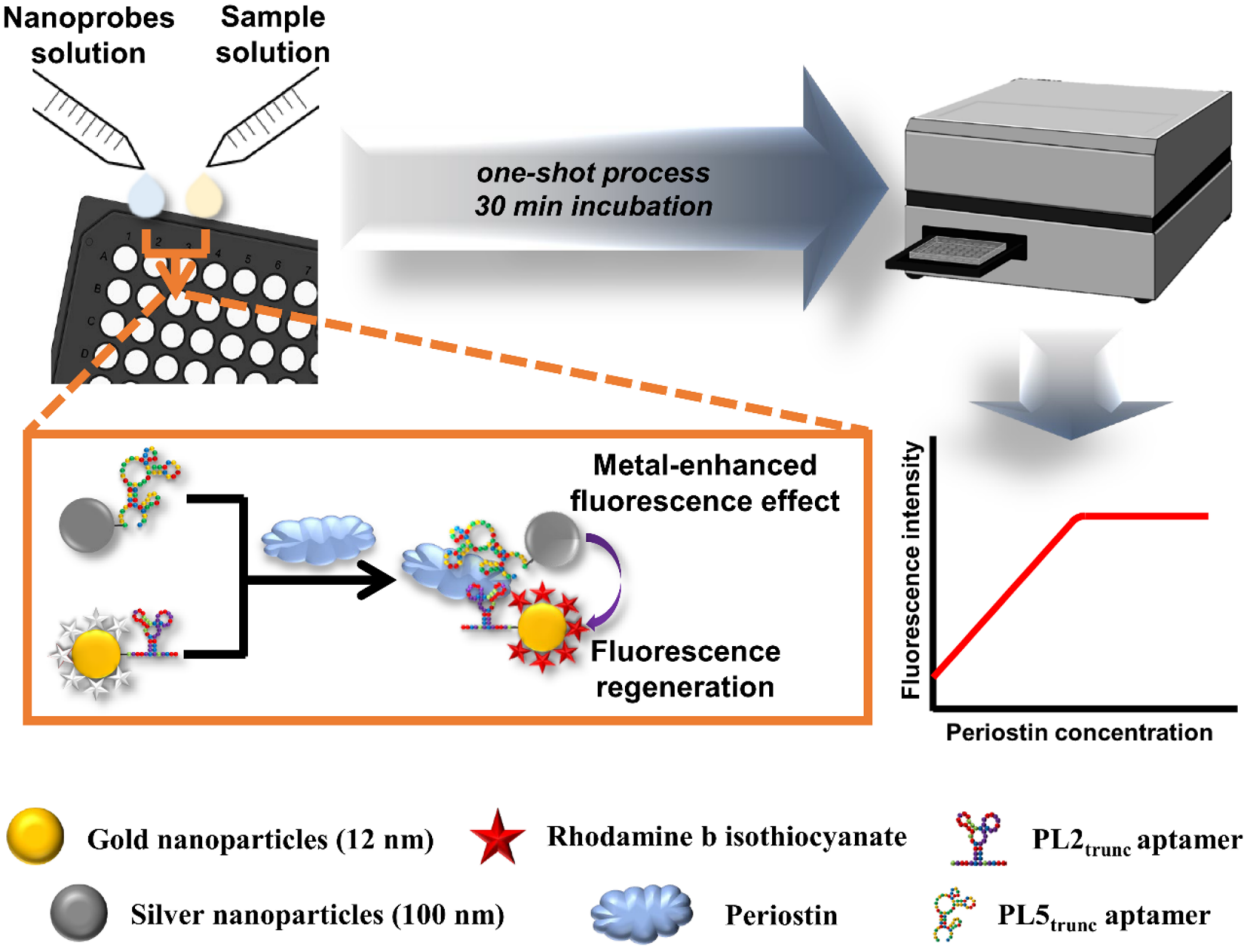
Detection strategy of periostin using the dual aptamer-based fluorescence nanosensors. The nanosensor uses Au nanoprobes constructed by RiTC, PL2_trunc_ aptamer, and Au nanoparticles, and Ag nanoprobes fabricated by the PL5_trunc_ aptamer and AgNPs to detect periostin without additional washing or signal amplification processes. The fluorescence of RiTC molecules is quenched by the FRET effect of AuNPs. In the presence of periostin, both nanoprobes interact with periostin to form a core-satellite structure, and the quenched fluorescence of Au nanoprobes is regenerated by the MEF effect of Ag nanoprobes due to the increased proximity of the two nanoprobes. Therefore, the nanosensor detects periostin through the regenerated fluorescence. Both figures of the microplate reader and 96-well black plate were created with BioRender.com (www.biorender.com).

In this study, we report the development of a rapid and label-free one-shot periostin detection platform with high sensitivity and selectivity using aptamers, rhodamine-b isothiocyanate (RiTC), and metal NPs (Fig. 1). By adding gold (Au) and silver (Ag) nanoprobes at once, the nanosensor provides rapid and direct detection of periostin without additional washing or signal amplification processes. For fabricating the nanosensor, we first developed two periostin-specific ssDNA aptamers (PL2_trunc_ and PL5_trunc_) and then applied them to fabricate the two nanoprobes. For synthesizing the Au nanoprobes, Au@RiTC NPs constructed by attaching RiTC to AuNPs and PL2_trunc_ aptamer were conjugated by Au-thiol interaction. Similarly, the Ag nanoprobes were synthesized by attaching the PL5_trunc_ aptamers to AgNPs by Ag-thiol interaction. The two aptamers allow the stable dispersion of both nanoprobes in solutions and provide high specificity for periostin. In addition, they play an essential role in increasing the proximity between the two nanoprobes because they have a relatively smaller size than antibodies and a simultaneous binding property to periostin. The fluorescence of RiTC on the AuNP surface is quenched by the FRET effect induced by the direct conjugation of RiTC and AuNPs, which decreases the noise of the platform. Moreover, in the presence of periostin, the two nanoprobes form core-satellite structures, which results in the fluorescence regeneration of the quenched RiTC by AuNPs through the MEF effect of AgNPs. Therefore, the two nanoprobes facilitate rapid one-shot detection of periostin without the need for any signal enhancement or washing processes. The proposed nanosensor successfully detected picomolar levels of periostin in buffer conditions within 30 min without additional signal amplification or washing processes. Furthermore, this platform exhibited possible clinical applicability by maintaining its detection performance, even in spiked human serum conditions. Therefore, by resolving the disadvantages of the existing detection methods, the proposed nanosensor provides a superior platform for convenient and rapid quantitative detection of periostin in real human samples. We expect the nanosensor to be a suitable alternative to antibody-based detection methods to identify patients with high periostin levels, and the platform will be helpful in future on-site medical applications because aptamers can be easily replaced with other detection probes.

## Results

### Development and characterization of the two periostin-specific ssDNA aptamers

**Figure 2:**
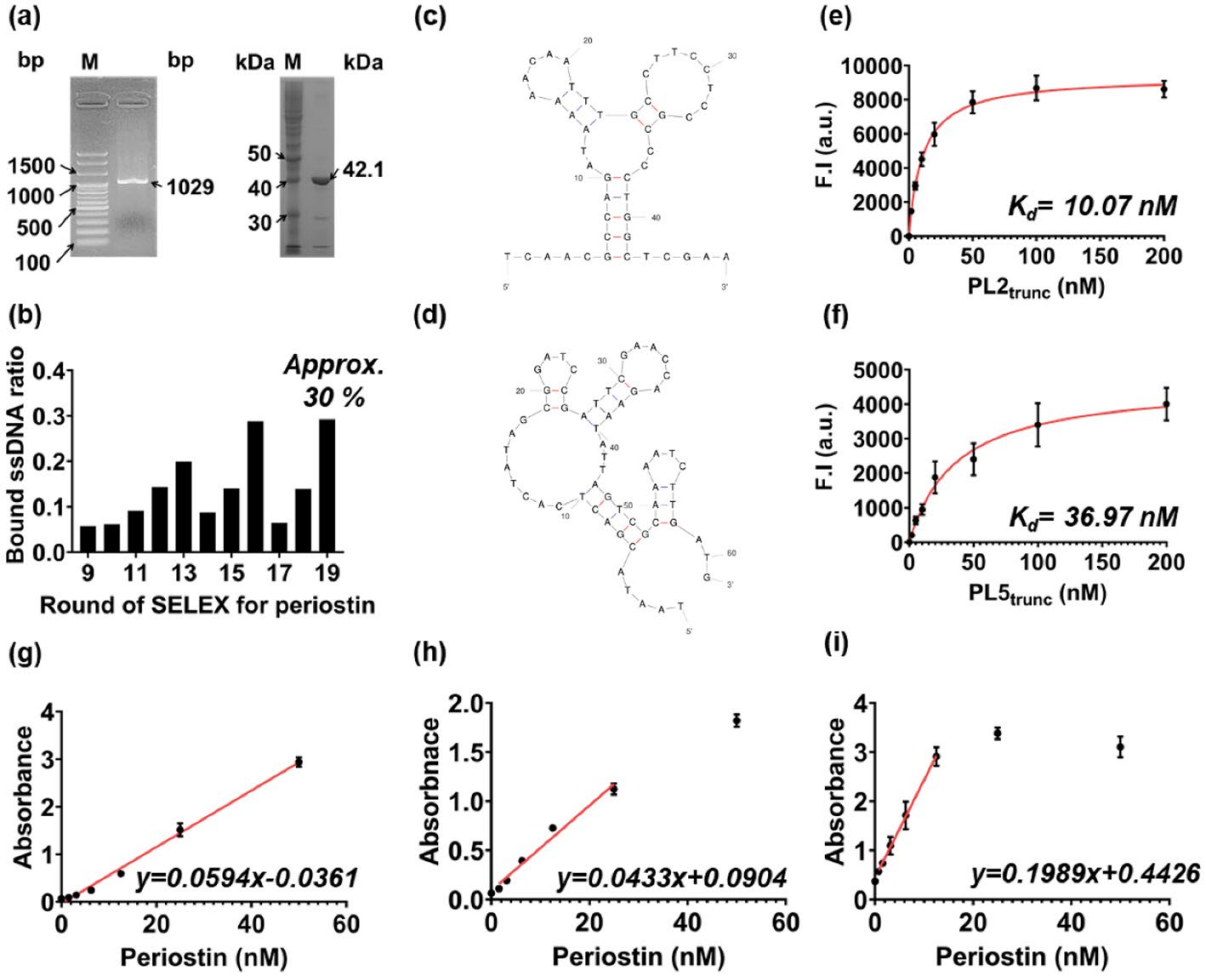
Development and characterization of the two periostin-specific ssDNA aptamers. (**a**) Gel electrophoresis of amplified periostin gene (1029 bp, left) and purified protein (42.1 kDa, right). The BioFACT™ 100 bp plus DNA ladder (left) and Step-view 10 kDa marker (right) were used as markers. Each original gel image is presented in Supplementary Fig. S1. M: marker, bp: base pair, kDa: kilodalton. (**b**) Bound ssDNA ratio for periostin in each round of SELEX. As rounds progressed, the percentage of bound ssDNA increased. However, in rounds 14 and 17, the bound ssDNA ratio decreased as the negative selection was performed. The last ssDNA library pool was sequenced after the bound ssDNA ratio repeatedly reached 30%. Predicted secondary structures of the (**c**) PL2_trunc_ and (**d**) PL5_trunc_ aptamers verified after the sequence optimization process. The structure and sequence of the two truncated aptamers were identical to some parts of the originals. The two structures were predicted using Mfold (http://www.unafold.org/mfold/applications/dna-folding-form.php). Fluorescence binding curves of the (**e**) PL2_trunc_ and (**f**) PL5_trunc_ aptamers for periostin. They showed a good binding affinity to periostin, with improved binding affinities compared to the original. The red line represents the saturation curve of aptamers for periostin. F.I.: fluorescence intensity, *K*_*d*_: affinity constant, a.u.: arbitrary unit, Bars: ± s.d., n = 3. The absorbance of direct ELONA 1 (**g**, dynamic range: 3.13–50 nM), direct ELONA 2 (**h**, dynamic range: 1.56–25 nM), and sandwich ELONA (**i**, dynamic range: 0.78–12.5 nM). Briefly, 1 μM of unmodified PL5_trunc_ aptamer and 0.2 μM of biotinylated PL2_trunc_ aptamer were directly applied to ELONA 1; 1 μM of unmodified PL2_trunc_ aptamer and 0.2 μM of biotinylated PL5_trunc_ aptamer were directly used for ELONA 2; 0.2 μM of amine-modified PL2_trunc_ aptamer and 1 μM of PL5_trunc_ aptamer were applied to sandwich ELONA. All ELONA systems exhibited a linear absorbance proportional to periostin concentration over the dynamic range. The red line represents the dynamic range of each ELONA. Bars: ± s.d., n = 3.

Periostin was purified using affinity and gel filtration chromatography after cloning and bacterial expression of the periostin (*POSTN*) gene (Fig. 2a and see Supplementary Fig. S1 online). Periostin-specific ssDNA aptamers were developed using a magnetic bead-based SELEX. An ssDNA library consisting of a central randomized region and primer regions at both ends for amplification was used in the first round of SELEX (see Supplementary Table S1 online). After each round of SELEX, ssDNA bound to periostin was collected, amplified by polymerase chain reaction (PCR), denatured in an alkaline solution to form a single strand, and then applied to the next round. The SELEX process was monitored via the bound ssDNA ratio, which was calculated by measuring the amount of unbound ssDNA and subtracting it from the amount in the applied library (Fig. 2b). The bound ssDNA ratio gradually increased as the rounds progressed, except for rounds 14 and 17 due to the negative selection; the negative selection was conducted whenever the bound ssDNA ratio was > 20% to remove non-specific bound ssDNA. The binding ratio repeatedly reached approximately 30% for rounds 16 and 19 after negative selection. We considered that effective selection was achieved after 19 rounds of SELEX based on a previous study wherein the aptamers were acquired after the bound ssDNA ratio repeatedly reached over 30%^25^.

After sequencing the last round pool, PL2 and PL5 aptamers were selected (see Supplementary Table S2 online). The predicted structures of both aptamers differed (see Supplementary Fig. S2a,b online). To determine the binding affinity of each aptamer, a magnetic bead-based fluorescence assay was performed using Cy3-labeled aptamers (see Supplementary Table S3 online). A saturation curve was applied to calculate the binding affinity (*K*_*d*_), and the *K*_*d*_ of the PL2 and PL5 aptamer was calculated to be 45.70 and 50.09 nM, respectively (see Supplementary Fig. S2c,d online). Aptamers may show an improved binding affinity for target molecules when the primer region is removed^26^. Therefore, each aptamer was sequence optimized by truncating the primer region based on the random sequence region and secondary structure of the original aptamers. The structures and sequences of the two truncated aptamers (Fig. 2c,d, and see Supplementary Table S2 online) were identical to the corresponding parts of their original structures and sequences (see Supplementary Table S2 and Fig. S2a,b online). In addition, the *K*_*d*_ of each truncated aptamer was also analyzed using the magnetic bead-based fluorescence assay with Cy3-labeled truncated aptamers (see Supplementary Table S3 online). As a result, they showed an improved *K*_*d*_ value (PL2_trunc_ *K*_*d*_ = 10.07 nM, PL5_trunc_ *K*_*d*_ = 36.97 nM) compared to the original aptamers (Fig. 2e,f). Therefore, the two truncated aptamers (hereafter referred to as PL2_trunc_ and PL5_trunc_ aptamers) were utilized for subsequent experiments.

### Verification of the simultaneous binding phenomenon of the two aptamers to periostin

We hypothesized that PL2_trunc_ and PL5_trunc_ aptamers would bind to different sites on periostin as their sequences and structures were highly different (Fig. 2c,d). Therefore, we performed two direct enzyme-linked oligonucleotide assays (ELONAs) to test our hypothesis. Briefly, 1 μM of unmodified PL5_trunc_ aptamer, together with 0.2 μM of biotinylated PL2_trunc_ aptamer, and vice-versa, were investigated in direct ELONA 1 and direct ELONA 2, respectively (see Supplementary Table S4 online). In both cases, the excessive unmodified aptamer and the biotinylated aptamer were sequentially added to a plate after attaching periostin to the microplate. As a result, both direct ELONAs exhibited linear absorbance proportional to periostin concentration over the dynamic range, although the unmodified aptamer was previously excessively reacted with periostin (Fig. 2g,h).

Furthermore, we performed a sandwich ELONA to verify whether the two aptamers could bind to periostin simultaneously or interact with each other without periostin. Amine-modified PL2_trunc_ and biotinylated PL5_trunc_ aptamers were used in the experiment as capture and detection aptamers, respectively (see Supplementary Table S4 online). After conjugating the amine-modified PL2_trunc_ aptamer to a microplate via an amide bond, it was sequentially reacted with periostin, the biotinylated PL5_trunc_ aptamer, streptavidin-poly horseradish peroxidase, and TMB solution. A linear absorbance, proportional to periostin concentration, was observed in the dynamic range (Fig. 2i), indicating that the two aptamers can bind to the target protein in a sandwich manner. Therefore, these results support that the two aptamers can simultaneously bind to periostin without interfering with each other, even though they exist in the same space. Based on these results, we exploited this phenomenon to control the proximity of the two nanoprobes in our fluorescence nanosensor.

### Synthesis of Au@RiTC NPs, Au nanoprobes, and Ag nanoprobes and verification of their physical properties

For detecting periostin, the Au and Ag nanoprobes were fabricated using aptamers, RiTC molecules, and metal NPs. The metal nanoprobes were synthesized by considering two important points: the quenching effect of the AuNPs and the optimal concentration of RiTC and aptamers. If the AuNPs cannot efficiently quench the fluorescence of the RiTC molecule, the nanosensor will not be able to detect periostin with a high noise ratio. Au nanoprobes fabricated with lower concentrations of RiTC causes weak fluorescence regeneration, which reduces the detection ability of the nanosensor. In addition, using low or very high amounts of aptamer also reduces the detection ability of the nanosensor due to the aggregation of the nanoprobe in solution or the steric-influenced inhibition of the binding with periostin, respectively.

**Figure 3:**
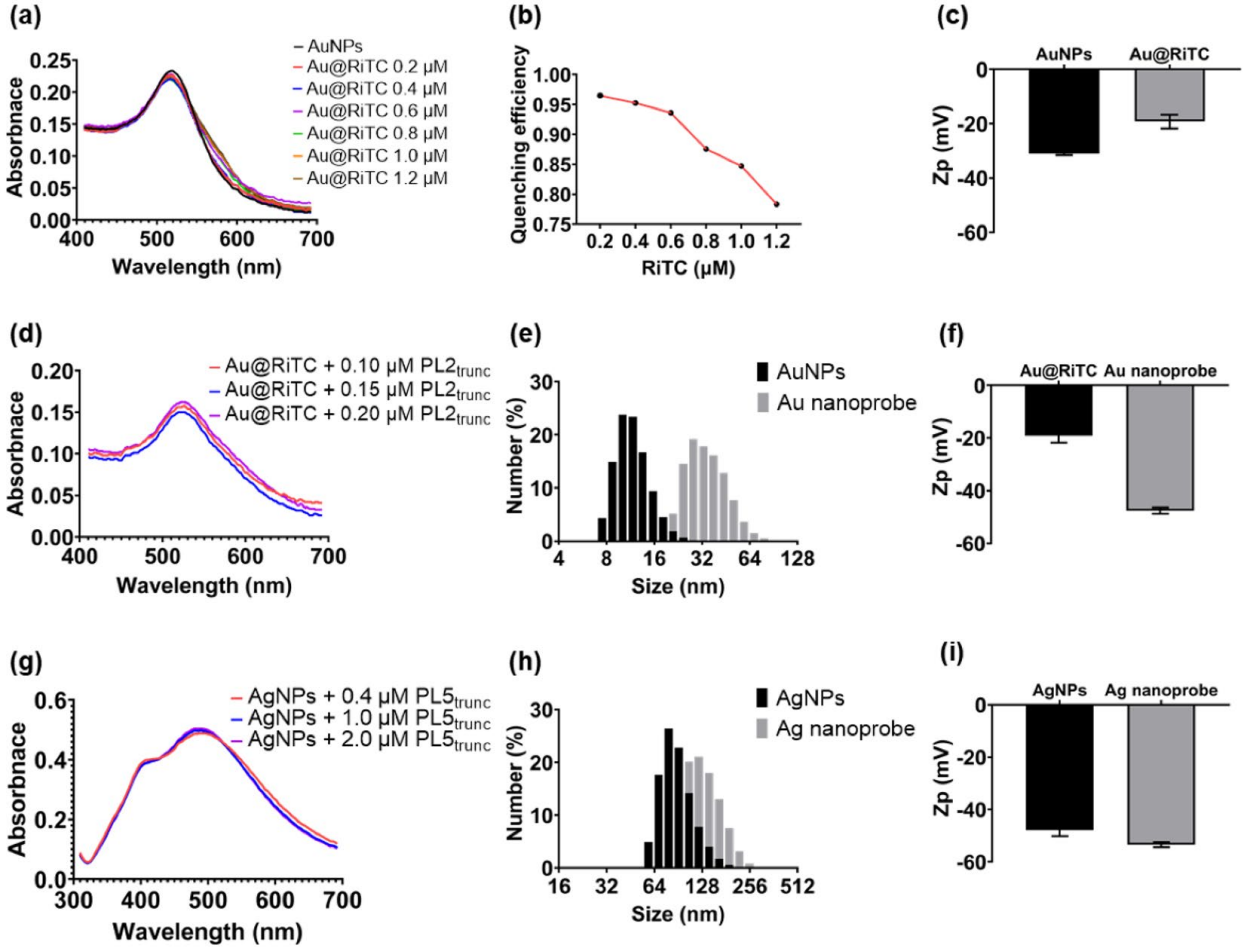
Verification of the physical properties of the Au@RiTC NPs, Au nanoprobes, and Ag nanoprobes. (**a**) UV–Vis spectra of AuNPs and various Au@RiTC NPs. The distinct UV–Vis spectrum of AuNPs was observed in all synthesized Au@RiTC NPs regardless of the RiTC concentration. (**b**) The quenching efficiency of AuNPs as the concentration of RiTC. The quenching efficiency of AuNPs was maintained over 90% up to 0.6 μM RiTC concentration, but decreased rapidly at subsequent concentrations. The red line represents the trend of quenching efficiency. (**c**) Zeta potential data of AuNPs and the synthesized Au@RiTC NPs. An increase in zeta potential was observed after the synthesis of Au@RiTC NPs. (**d**) UV–Vis spectra of the synthesized Au nanoprobes synthesized with various concentrations of aptamers. All synthesized Au nanoprobes exhibited the unique spectrum of AuNPs. (**e**) DLS data of AuNPs and the synthesized Au nanoprobes. The size of Au nanoprobes was significantly shifted to the right after the aptamer was bound to the Au@RiTC NPs. (**f**) Zeta potential data of Au@RiTC NPs and the synthesized Au nanoprobes. The synthesized Au nanoprobes exhibited significantly decreased zeta potential after the attachment of the aptamer. (**g**) UV–Vis spectra of the synthesized Ag nanoprobes reacted with various concentrations of the aptamers. All synthesized Ag nanoprobes showed a unique spectrum of AgNPs. (**h**) DLS data of AgNPs and the synthesized Ag nanoprobes. The size of the Ag nanoprobes shifted to the right after the attachment of the aptamers to AgNPs. (**i**) Zeta potential data of AgNPs and the synthesized Ag nanoprobes. The synthesized Ag nanoprobes showed decreased zeta potential due to the conjugation of the aptamers. Zp: zeta potential, Bars: ± s.d., n = 3.

Au nanoprobes were synthesized via two processes, namely the construction of Au@RiTC NPs using RiTC and AuNPs and attaching of PL2_trunc_ aptamers to Au@RiTC NPs. To synthesize Au@RiTC NPs, various concentrations of RiTC (0.2–1.2 μM) were added to 1.0 nM of AuNPs based on a previous study^27^. RiTC molecules have an excitation wavelength of 530 nm and an emission wavelength of 580 nm and can be spontaneously attached to AuNPs through conjugation between the sulfur atoms of the isothiocyanate (ITC) group and the AuNPs^28^. All synthesized Au@RiTC NPs exhibited almost identical absorption spectra to AuNPs (minor broadening was observed in the longer wavelength) regardless of the RiTC concentration (Fig. 3a). The core size of the synthesized Au@RiTC NPs was 12 nm, identical to that of AuNPs (see Supplementary Fig. S3a,b online), and the synthesized Au@RiTC NPs exhibited no aggregated morphology in solution. Next, the quenching effect of AuNPs was investigated. Free RiTC solutions were strongly fluorescent, and the intensity increased linearly as the RiTC concentration increased. However, Au@RiTC NPs were poorly fluorescent, and the intensity was increased only when RiTC was added at 0.8 μM or more (see Supplementary Fig. S3c,d online). Based on this, the quenching efficiency of AuNPs was calculated as follows: (F.I_RiTC_–F.I_Au@RiTC NPs_)/F.I_RiTC_. The AuNPs exhibited > 90% quenching efficiency at RiTC concentrations below 0.6 μM, but a decreased efficiency at RiTC concentrations above 0.8 μM (Fig. 3b). From this result, we speculated that the surface of AuNPs could be saturated by RiTC at 0.6 μM. Therefore, a RiTC concentration of 0.6 μM was used for the synthesis of Au@RiTC NPs to maximize fluorescence quenching and, hence, the detection performance. In addition, we investigated the stability and reproducibility of the synthesized Au@RiTC NPs by analyzing the TEM images and UV–Vis spectra and calculating the quenching efficiency. We first investigated the stability and quenching efficiency of the Au@RiTC NPs one week after synthesis. The synthesized Au@RiTC NPs showed a 12 nm core size and a unique spectrum of AuNPs, which were maintained one week after synthesis (see Supplementary Fig. S4a–d online). Additionally, the synthesized Au@RiTC NPs showed consistent quenching efficiency of > 90% at one week (see Supplementary Fig. S4e online). Next, the reproducibility of the Au@RiTC NPs was verified by synthesizing them in different batches and comparing their TEM images, UV–Vis spectra, and quenching efficiencies. All synthesized Au@RiTC NPs exhibited a 12 nm core size and a unique UV–Vis spectrum of AuNPs, regardless of the batch (see Supplementary Fig. S5a–d online). In addition, the quenching efficiency also showed a constant efficiency of over 90% in all batches (see Supplementary Fig. S5e online). Therefore, these results indicate that the synthesized Au@RiTC NPs have stable particle dispersion, constant quenching efficiency, and no batch-to-batch variation even when synthesized repeatedly. However, the hydrodynamic radius of the synthesized Au@RiTC NPs could not be measured because they were aggregated after the centrifugation process, turning from red to dark violet (see Supplementary Fig. S6a online). We speculated that the aggregation of Au@RiTC NPs during high centrifugation was due to the reduced repulsive force by the attachment of RiTC to the AuNPs. Indeed, the Au@RiTC NPs showed a higher zeta potential than the AuNPs, supporting that the repulsive force between the Au@RiTC NPs is markedly reduced due to RiTC (Fig. 3c). Therefore, the fabricated Au@RiTC NPs were utilized to synthesize Au nanoprobes without centrifugation. Next, Au@RiTC NPs were reacted with different concentrations of thiolated PL2_trunc_ aptamers (0.05–0.20 μM) (see Supplementary Table S5 online), and purified by centrifugation to synthesize the Au nanoprobes. All synthesized Au nanoprobes showed a distinct red solution due to the SPR effect^29^, except the one that was reacted with 0.05 μM PL2_trunc_ aptamer (see Supplementary Fig. S6b online). It seems that Au nanoprobes synthesized using 0.05 μM PL2_trunc_ aptamer undergo aggregation during centrifugation due to weaker stability than the others. Indeed, we synthesized the Au nanoprobes using 0.10 μM of thiolated aptamers labeled with Cy3 at the 3′-end, measured the fluorescence intensity of the supernatant after centrifugation, and calculated the concentration of bounded aptamers used for binding as follows: bound aptamer (%) = ((F_applied aptamers_ − F_supernatant_) / F_applied aptamers_) × 100. As a result, the bound aptamer was calculated as 70%, which means that more than 0.07 μM aptamer is required by the 1 nM Au@RiTC NPs to synthesize the Au nanoprobe. Therefore, the Au nanoprobes were fabricated by adding 0.1 μM of aptamers. Meanwhile, we investigated the physical properties of the synthesized Au nanoprobes. The core size was 12 nm, the same as AuNPs (see Supplementary Fig. S7a,b online). In addition, all synthesized Au nanoprobes showed the unique absorption spectrum of AuNP regardless of the concentration of the aptamer, indicating that they exhibited stable dispersion after the synthesis (Fig. 3d and see Supplementary Fig. S7c online). Moreover, the size and zeta potential were significantly changed after the synthesis of the Au nanoprobe (Fig. 3e,f). These results support that the PL2_trunc_ aptamers were successfully conjugated to the Au@RiTC NPs.

To fabricate Ag nanoprobes, different concentrations of the thiolated PL5_trunc_ aptamer (0.4–2.0 μM) (see Supplementary Table S5 online) were reacted with 2 pM of AgNPs and purified by centrifugation. As a result, the size of the synthesized Ag nanoprobe was 100 nm, the same as that of AgNPs, as determined from TEM images (see Supplementary Fig. S8a,b online). In addition, all synthesized Ag nanoprobes exhibited the distinct absorption spectrum of AgNPs, regardless of the PL5_trunc_ aptamer concentration (Fig. 3g and see Supplementary Fig. S8c online). More than 200,000 times (or greater) aptamer concentration is required to react with 100 nm-sized metal NPs^30^. Indeed, we synthesized Ag nanoprobes using 0.40 µM of thiolated aptamers labeled with Cy3 at the 3′-end and then calculated the concentration of the attached aptamer. The bound aptamer was calculated to be 85% (0.34 μM), which is similar to the reported result. Therefore, 0.4 μM of the PL5_trunc_ aptamer was utilized to construct the Ag nanoprobes. Meanwhile, the Ag nanoprobe showed a change in the size and a negative zeta potential after the synthesis of the Ag nanoprobes (Fig. 3h,i). These results support that the PL5_trunc_ aptamers were successfully conjugated to the AgNPs.

### Feasibility test and optimization of the fluorescence nanosensors for the detection of periostin

The nanosensor detects periostin by combining the simultaneous binding property of aptamers and the FRET and MEF effects of metal nanoparticles. When periostin is present in the solution, the two nanoprobes can bind to the target protein simultaneously due to the aptamer-protein interactions and form a core-satellite structure (Fig. 1). At this time, the proximity between the two nanoprobes significantly increases, allowing the MEF effect of the Ag nanoprobes to restore the quenched fluorescence of the RiTC attached on the Au nanoprobes. Therefore, the nanosensor can detect periostin through the regenerated fluorescence intensity. However, if fluorescence regeneration is not affected by the simultaneous aptamer-periostin binding, the fluorescence will regenerate because their proximity is significantly increased by large amounts of the two nanoprobes present in solutions, even in the absence of periostin. In addition, if the fluorescence regeneration is not affected by the increased proximity of the two nanoprobes, the fluorescence regeneration will not occur even if periostin is present in the solution. Therefore, a screening test was performed using the two synthesized nanoprobes to verify that the fluorescence was regenerated due to the proximity induced by the interaction between periostin and the two nanoprobes.

**Figure 4:**
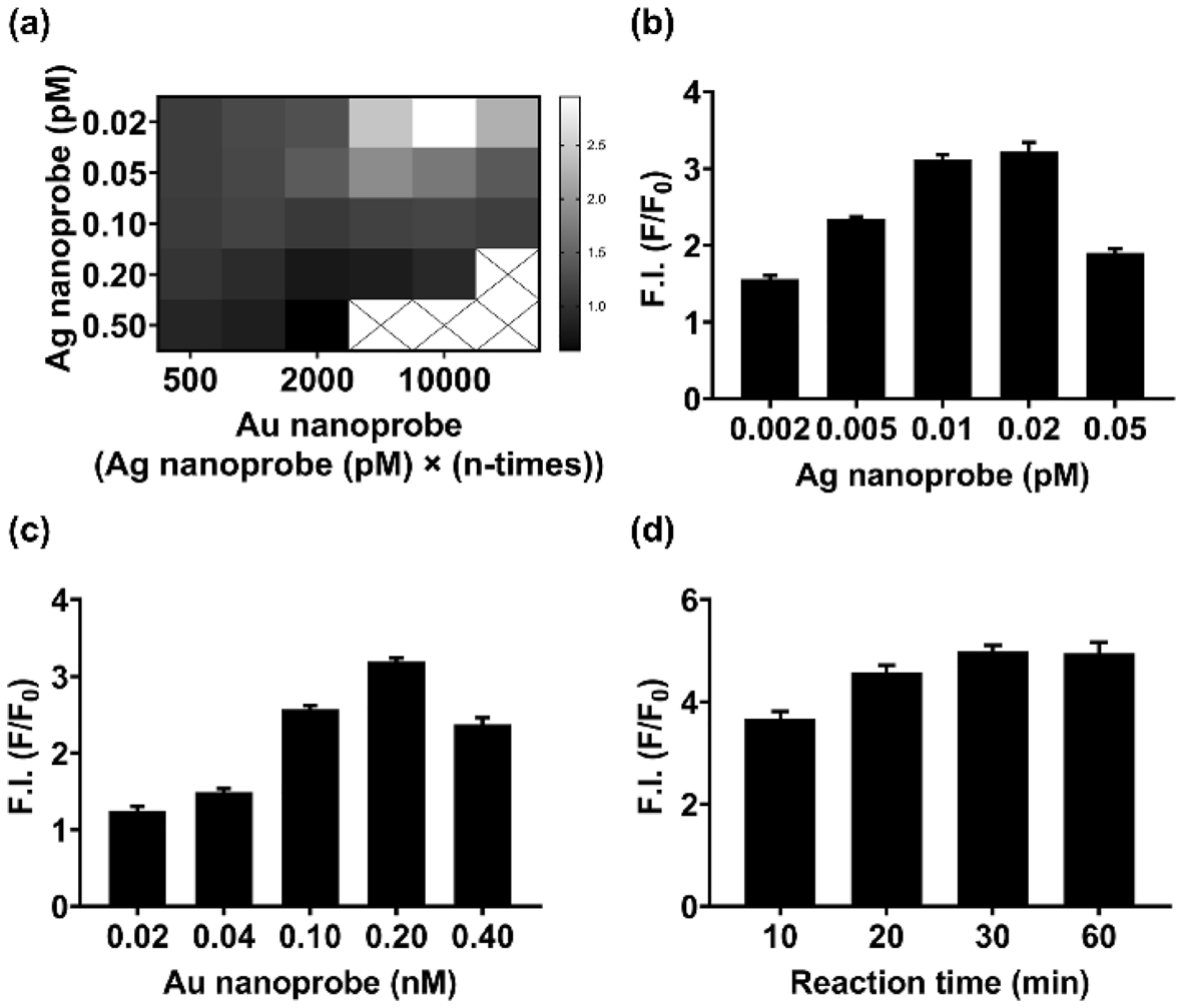
Feasibility test and optimization of the fluorescence nanosensor for the detection of periostin. (**a**) Heat map of the nanosensor after reacting various concentrations of Ag and Au nanoprobes with periostin. Meaningful fluorescence intensity was observed at specific concentration ranges of both Ag nanoprobes and Au nanoprobes, and the maximum fluorescence intensity was observed with 0.02 pM Ag nanoprobes and 0.2 nM Au nanoprobes (10,000 times higher than the concentration of 0.02 pM Ag nanoprobes). White and black represent the highest and lowest fluorescence intensity, respectively. (**b**) Fluorescence intensity of the nanosensor when various concentrations of Ag nanoprobes and 0.2 nM Au nanoprobes were reacted with periostin. The highest fluorescence intensity was observed with 0.02 pM Ag nanoprobes. (**c**) Fluorescence intensity of the nanosensor when different concentrations of Au nanoprobes and 0.02 pM Ag nanoprobes were reacted with periostin. The fluorescence intensity was maximum with 0.2 nM Au nanoprobes. (**d**) Fluorescence intensity of the nanosensor following various reaction times with periostin. Meaningful fluorescence intensity was observed at 10 min, and the fluorescence intensity was saturated after 30 min. F.I.: fluorescence intensity, F/F_0_: the value obtained by dividing the fluorescence intensity of the sample (F) by the fluorescence intensity of the blank (F_0_), Bars: ± s.d., n = 3.

For this, various concentrations of Ag and Au nanoprobes were reacted in the absence and presence of 10 nM periostin; Ag nanoprobes were tested at concentrations of 0.02, 0.05, 0.1, and 0.5 pM and the Au nanoprobes at amounts of 500, 1,000, 2,000, 5,000, 10,000, and 20,000 times more than the Ag nanoprobe concentration. Thereafter, we monitored the relative fluorescence regeneration of the nanosensor (F/F_0_). The fluorescence regeneration of the nanosensor occurred at specific concentration ranges of both nanoprobes, and the highest F/F_0_ appeared when Ag nanoprobes with a concentration of 0.02 pM and Au nanoprobes with a concentration 10,000 times higher than that of the Ag nanoprobes were reacted (Fig. 4a). In addition, the fluorescence of the nanosensor was regenerated only when periostin was present in solution, and no continuous fluorescence regeneration (irrespective of the presence or absence of periostin) or quenching (in the presence of periostin) was observed (see Supplementary Fig. S9 online). These results support that the fluorescence regeneration of the nanosensor is dependent only on the proximity of the two nanoprobes caused by simultaneous aptamer-periostin binding. Moreover, it indicates that periostin can be quantified by measuring the regenerated fluorescence intensity of the nanosensor.

We also cross-checked whether the combination of 0.02 pM Ag nanoprobes and 0.2 nM Au nanoprobes exhibited maximal fluorescence regeneration by performing the two experiments: verification of fluorescence intensity when various concentrations of Ag nanoprobes were applied to 0.2 nM Au nanoprobes and when various concentrations of Au nanoprobes were applied to 0.02 pM Ag nanoprobes. The maximum fluorescence regeneration was observed at 0.02 pM and 0.2 nM of the Ag and Au nanoprobes, respectively (Fig. 4b,c). Furthermore, we verified whether the MEF effect of Ag nanoprobes practically contributes to regenerating the quenched fluorescence of the nanosensor. This was assessed by treating periostin respectively with Ag nanoprobes, Au nanoprobes, or both nanoprobes. Fluorescence regeneration was highest in the presence of both nanoprobes (see Supplementary Fig. S10 online), suggesting that fluorescence regeneration and enhancement could occur by the integrated MEF effect of both nanoprobes and, in particular, the MEF effect of the Ag nanoprobe significantly contributed to the fluorescence regeneration of RiTC^31^.

Next, the reaction time of the nanosensor was investigated using the optimized concentration of each nanoprobe. Significant fluorescence intensity was observed 10 min after adding both nanoprobes, and the signal was saturated after 30 min (Fig. 4d). Therefore, 0.02 pM of Ag nanoprobe, 0.20 nM of Au nanoprobe, and 30 min of reaction time were set as the optimal parameters for the efficient detection of periostin.

Lastly, we investigated the effect of AgNPs of different sizes on periostin detection (see Supplementary Fig. S11 online). We synthesized Ag nanoprobes using 40 nm AgNPs (group 1) and compared them with the fluorescence intensity of the nanosensor used the Ag nanoprobes synthesized with 100 nm AgNPs (group 2) while applying them to periostin detection. Although a significant fluorescence intensity was observed in group 1, a further increased fluorescence intensity was observed in group 2. We believe the increased fluorescence may be due to the following two reasons: large metal NPs have a higher scattering-absorption effect than small metal NPs, which is advantageous for metal-enhanced fluorescence^32^. In addition, a large amount of target protein and gold nanoprobes can react with one Ag nanoprobe because large-sized metal nanoparticles have a large surface area; thus, this effect results in a signal amplification effect. Therefore, we applied the Ag nanoprobes synthesized with 100 nm AgNP to detect periostin.

### Performance test of the fluorescence nanosensor in buffer

**Figure 5:**
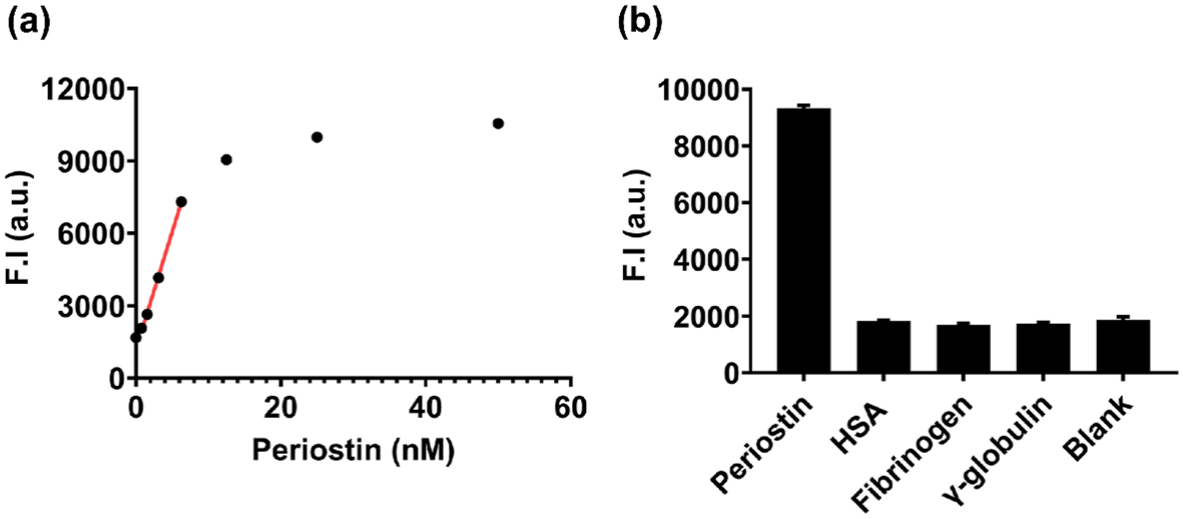
Verification of the detection performance of the fluorescence nanosensor. (**a**) Sensitivity test of the nanosensor in buffer conditions. Two-fold serial dilutions of periostin solutions (from 50 nM) were treated with the nanosensor. The red line represents the dynamic range of the nanosensor in buffer conditions. The linear dynamic range was from 0.78 to 6.25 nM in buffer condition, the calculated detection limit was 106.04 pM, and the computed CV and recovery rate were 1.51% and 94.87 to 114.10%, respectively. (**b**) Specificity test of the nanosensor in buffer conditions. 10 nM of periostin and 100 nM of the other proteins were reacted. The highest fluorescence intensity was observed only in periostin. F.I.: fluorescence intensity, a.u.: arbitrary unit, Bars: ± s.d., n = 3.

Next, we verified the detection performance of the nanosensor in buffer conditions. The sensitivity of the nanosensor was investigated using the binding buffer containing two-fold serial dilutions of periostin (from 50 nM) (Fig. 5a). As a result, the fluorescence intensity was proportional to the periostin concentration in the dynamic range (0.78–6.25 nM, *R*^*2*^ = 0.999), and the calculated LOD was 106.04 pM. This was similar to the dynamic range (0.13–4.00 nM) observed with the sandwich ELISA method^11^. The average CV was calculated as 1.51% (see Supplementary Table S6a online), indicating significant reproducibility. The calculated recovery rate was 94.87–114.10% (see Supplementary Table S6b online). Meanwhile, the stability and reproducibility of the established nanosensor were investigated via verification of the detection performance up to half a month after synthesis of the two nanoprobes and confirmation of the dynamic range reproducibility in different batches. As a result, the nanosensor showed similar fluorescence intensities regardless of the time point of the measurement (see Supplementary Fig. S12a online) and exhibited high reproducibility of the dynamic range across different batches (see Supplementary Fig. S12b online). In addition, the specificity of the nanosensor toward periostin was verified using HSA, fibrinogen, and γ-globulin, the three most abundant proteins in human blood^33^. For the specificity test, 10 nM of periostin and 100 nM of the test proteins were subjected to the assay condition (Fig. 5b). Significant fluorescence intensity change was observed only in the presence of periostin, indicating excellent selectivity of the nanosensor toward periostin.

Thus, the nanosensor shows comparable detection performance to sandwich ELISA, while it needs only 30 min of detection time and requires no additional washing or signal amplification steps (Table 1). Unlike the conventional methods, this platform provides a simple, rapid, low-cost, point-of-care method for the one-shot detection of periostin.

**Table 1:** Comparison of the proposed nanosensor with other conventional periostin detection methods.

Ref	Assay type	Transducing method	Recognition probe	Several washing processes	Pretreatment process for signal amplification	Signal amplification process	Detection time	Detection limit (pM)	Usability
^10^	Sandwich	Fluorescence	Monoclonal Antibody	Needed	Needed	Needed	7 h 30 min	–	Expert required
^11^	Sandwich	Absorbance	Monoclonal Antibody	Needed	Needed	Needed	5 h 30 min	20	Expert required
This work	Sandwich	Fluorescence	Aptamer	Not needed	Not needed	Not needed	30 min	106.04	Highly accessible

Till now, several conventional periostin detection methods have been developed using antibodies in a sandwich manner. In 2003, Sasaki et al.^10^ reported a chemiluminescence-based assay system that used periostin as a marker for detecting preeclampsia in normal pregnancy. Recently, Gardernaier et al.^11^ also reported an absorbance-based system, which was employed to assess periostin levels in the blood of patients with asthma and cardiovascular disease. The methods, however, use two monoclonal antibodies-based sandwich ELISA system, which requires technical expertise for operation, complicated procedures, and high costs. Additionally, these methods require washing, signal amplification, and strict storage of materials, all of which limit their practical application. Most importantly, both methods require approximately six hours for analysis, which makes them unsuitable for point-of-care detection of periostin. In this context, there is an urgent need for a rapid, label-free, sensitive platform for periostin detection.

### Verification of the clinical applicability of the fluorescence nanosensor

**Figure 6:**
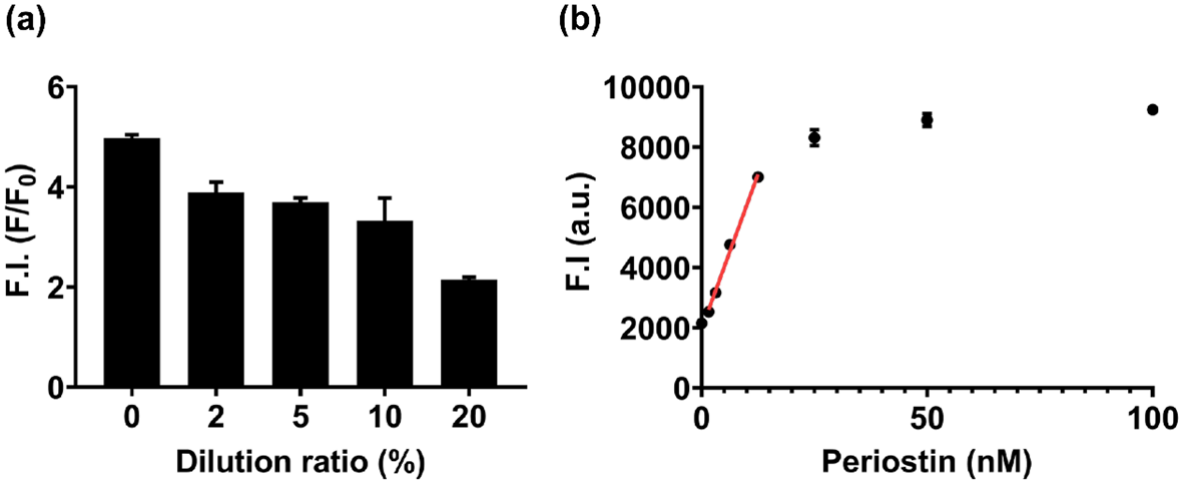
Clinical applicability test of the fluorescence nanosensor to detect periostin. (**a**) Fluorescence intensity of the nanosensor in various diluted human serum conditions. The fluorescence intensity of the nanosensor began to decrease gradually when human serum was diluted from 2 to 10%, and became significantly low at a dilution of 20%. F/F_0_ represents the value obtained by dividing the fluorescence intensity of the sample (F) by the fluorescence intensity of the blank (F_0_). (**b**) Sensitivity test of the fluorescence nanosensor in spiked human serum conditions, wherein the human serum was ten-fold diluted. Two-fold serial dilutions of periostin solutions (from 100 nM) were reacted. The red line represents the dynamic range of the nanosensor in serum-spiked conditions. The linear dynamic range was from 1.56 to 12.5 nM in buffer condition, the calculated detection limit was 463.3 pM, and the computed CV and recovery rate were 1.71% and 89.10–108.96%, respectively. F.I.: fluorescence intensity, a.u.: arbitrary unit, Bars: ± s.d., n = 3.

Serum periostin levels are elevated in patients with various diseases and are considered a marker of the disease severity^7–9^. To evaluate the feasibility of the clinical application of the nanosensor, we investigated the detection performance of the nanosensor in spiked human serum conditions. We first investigated the dilution ratio of human serum to verify whether the detection performance of the nanosensor varies with the dilution ratio of human serum. Human serum was diluted with various ratios, and periostin was spiked into the solution. As a result, the fluorescence intensity of the nanosensor began to decrease along with the dilution ratio of the human serum, and it was significantly reduced at 20% (v/v) dilution of human serum (Fig. 6a). We speculated that the matrix effect of human serum affected the detection performance of the nanosensor in two ways: the increase in the blank signal due to increased autofluorescence effect of serum and the inhibition of aptamer-periostin binding by high concentrations of biomolecules^34^. Indeed, the fluorescence intensity of the nanosensor gradually decreased with the serum dilution ratio, and the blank signal was markedly increased in 20% diluted human serum solution (see Supplementary Fig. S13online). Therefore, a 10% dilution ratio was selected in consideration of the decrease in fluorescence intensity and the change in the blank signal of the nanosensor according to the serum dilution ratio.

Next, a two-fold serial dilution of periostin (from 100 nM) was spiked in a 10% diluted human serum solution and then allowed to react with the nanosensor. As a result, the fluorescence intensity showed linearity with the concentration of periostin in the dynamic range (1.5–12.5 nM *R*^*2*^ = 0.993), and the calculated detection limit was 463.3 pM (Fig. 6b). The average CV was calculated as 1.71% and the recovery rates were between 89.10 and 108.96% (see Supplementary Table S7 online). Serum periostin levels in patients with various diseases range from 4.8 to 21.5 nM and increase with disease severity (Table 2). The detection limit of our nanosensor is approximately 4.63 nM based on 100% human serum, suggesting that the nanosensor can be used to discriminate between healthy individuals and patients with various diseases and classify the disease severity. Therefore, the proposed nanosensor can be applied to actual human serum and provide a unique platform for disease diagnosis and prognosis by enabling rapid and label-free detection of periostin.

**Table 2:** Comparison of cut-off values and disease severity according to blood periostin levels in various diseases.

Disease	Specimen	Cut-off value and disease severity by periostin concentration in the blood		Refs.
Non-small cell lung cancer (NSCLC)	Serum	Cut-off value	4.8 nM	^7^
		Advanced NSCLC	6.1 nM	
Obstructive sleep Apnea-hypopnea syndrome (OSAHS)	Serum	Cut-off value	4.2 nM	^8^
		Moderate	4.9 nM	
		Severe	7.0 nM	
Diabetic retinopathy (DR)	Serum	Cut-off value	18.6 nM	^9^
		Type 2diabetes mellitus w/o DR	14.9 nM	
		Type 2diabetes mellitus with DR	20.7 nM	
		Non-proliferative DR	20.1 nM	
		Proliferative DR	21.5 nM	

## Discussion

We developed a novel one-shot dual aptamer-based fluorescence nanosensor using two aptamer-based nanoprobes to detect periostin. The proposed nanosensor provides a convenient and practical method that is rapid, label-free, and sensitive for the detection of periostin using two metal nanoprobes without any signal amplification or washing process. The nanosensor exhibited reliable detection performance at the picomolar level within 30 min in both buffer and spiked human serum conditions through the following properties of the two aptamers and metal nanoparticles: high target specificity of the two aptamers and their simultaneous binding property to periostin and the high surface area-to-volume ratio of metal NPs and their ability to quench and regenerate fluorescence with distance. The nanosensor achieved a detection limit similar to other periostin assay systems while improving several challenges using low-cost materials, such as aptamers, nanoparticles, and fluorophores. Moreover, it provides the possibility for constructing a scalable platform for detecting various blood biomarkers because aptamers attached to nanoparticles can be easily replaced with other detection probes using metal-thiol interactions. Therefore, the proposed sensor is user-friendly, inexpensive, and easily accessible and shows strong potential to detect high levels of periostin. Although the proposed nanosensor has some limitations, such as still requiring serum-type samples in the detection and being usable only in diseases with high periostin concentrations, we believe that our nanosensor will be a suitable alternative to antibody-based detection methods to identify patients with high periostin levels and that this platform will be widely applied to various on-site medical diagnoses.

## Materials and methods

### Materials

The periostin *(POSTN)* gene (transcript variant 1 mRNA, NM_006475.2) was synthesized by Bioneer Inc. (Daejeon, Republic of Korea). The BL21 (DE3) cells, pET-28a vectors, and pENTR™/D-TOPO® cloning kit were purchased from Invitrogen (Waltham, MA, USA). Isopropyl-β-D-thiogalactopyranoside (IPTG) was purchased from Merck (Hessen, Germany). The Hi-trap Ni-NTA affinity column and gel filtration column packed with Sephadex G-25 superfine were obtained from GE Healthcare (New York, NY, USA). Nucleospin Gel and a PCR Clean-up kit were purchased from MACHEREY-NAGEL (Düren, Germany). Dynabeads™ His-Tag Isolation and Pulldown beads, Dynabeads™ MyOne™ Streptavidin C1 beads, a Pierce™ TMB substrate kit, Pierce™ Streptavidin Poly-horseradish peroxidase (HRP), and Pierce™ Maleic Anhydride Activated Plates were bought from Thermo Scientific (Waltham, MA, USA). All oligonucleotides were synthesized by Bionics Inc. (Seoul, Republic of Korea). Gold (III) chloride trihydrate, Tris(2-carboxyethyl) phosphine-hydrochloride (TCEP-HCl), silver nanoparticles (100 nm), bovine serum albumin (BSA), human serum albumin (HSA), fibrinogen, γ-globulin, and human serum (human male AB plasma, heat-inactivated) were purchased from Sigma-Aldrich (Saint Louis, MO, USA). RiTC was purchased from Cayman Chemicals (Ann Arbor, MI, USA). A 96-well black immunoplate was purchased from SPL Life Sciences Co., Ltd. (Pocheon, Republic of Korea).

### Instruments

Ultraviolet–visible (UV–Vis) and fluorescence spectra were obtained using an Evolution 300 UV–Vis spectrophotometer (Thermo Scientific, Waltham, MA, USA) and an RF-6000 spectrofluorometer (Shimadzu, Kyoto, Japan). Transmission electron microscopy (TEM) images were collected using a JEM-2100 (LaB6) microscope (JEOL, Tokyo, Japan). Dynamic light scattering (DLS) and zeta potential of the nanoparticles were measured using a Zetasizer Nano apparatus (Malvern Panalytical Ltd., Malvern, UK). Fluorescence intensity was measured with a Synergy™ HT multi-detection microplate reader (BioTek, Winooski, VT, USA). The absorbance of the ssDNA library was measured using a UV–Vis spectrophotometer (Biochrome Ltd., Cambridgeshire, UK).

### Cloning, expression, and purification of periostin

Recombinant periostin was expressed according to a previously reported method^35^. Briefly, the periostin (*POSTN*) gene was amplified using forward primer (5′-CCCGGATCCGAAAAATCTCTGCACGAAAAACTG-3′) and reverse primer (5′-CCCGAGCTCTCACTGAGAACGACCTTCACGCAGACG-3′). The amplified gene was cloned into a pET-28a vector containing a 6 × His affinity tag, and the plasmid was inserted into BL21 (DE3) cells. The cells were induced by adding 0.1 mM IPTG, incubated for 6 h at 37 °C, and then harvested. The harvested cells were lysed using a lysis buffer (20 mM Tris–HCl [pH 8.0], 500 mM NaCl, 0.5 mM β-mercaptoethanol, 10 mM imidazole, and 5% [v/v] glycerol) and then sonicated on ice. The supernatant was separated from the inclusion bodies via centrifugation (4 °C, 18,000 rpm, 40 min) and filtered using a 0.22-μm bottle-top filter. The supernatant was then loaded onto a Hi-trap Ni-NTA affinity column and further purified using a gel filtration column packed with Sephadex G-25 superfine resin. The purified periostin solution was concentrated, aliquoted, and stored at − 80 °C until further use.

### In vitro selection and sequence optimization of the PL2 and PL5 aptamers

PL2 and PL5 aptamers specifically bound to periostin were developed using a magnetic bead-based systematic evolution of ligands by exponential enrichment (SELEX). The SELEX process consists of positive and negative selection. Negative selections were performed together with positive selections whenever the binding ratio of the previous round was 20% or higher. The ssDNA library consists of three parts; a central region of 40 random nucleotides, a forward primer region, and a reverse primer region. All sequences used in the SELEX are listed in Table S1.

In each positive round of SELEX, 100 pmol of ssDNA library solution was prepared in binding buffer (20 mM Tris-HCl [pH 8.0], 100 mM NaCl, 5 mM KCl, 5 mM MgCl_2_, and 0.05% [v/v] Tween 20), heated in boiling water for 5 min, and then cooled on ice for 1 h to allow spontaneous secondary structures to form. To prepare the periostin-immobilized magnetic beads complex, 14 μL of His-tag Isolation and Pulldown magnetic beads were reacted with 500 pmol of His-tagged periostin for 1 h at 25 °C in the binding buffer. The bead-protein complexes were reacted with the ssDNA library solution for 1 h at 25 °C following washing twice with binding buffer. After separating the bead complexes using an external magnetic bar, the unbound ssDNA solution was collected, and the absorbance was measured at 260 nm to calculate the amount of unbound ssDNAs. After washing the bead complexes twice with the binding buffer, the periostin-bound ssDNAs complex was eluted using binding buffer containing 500 mM imidazole. After purifying with an ethanol precipitation process, bound ssDNAs were amplified via a PCR process with a biotinylated reverse primer and forward primer (Table S1). PCR conditions were as follows: (a) 98 °C for 150 s, (b) 98 °C for 30 s, (c) 55 °C for 30 s, (d) 72 °C for 30 s, (e) repeating steps of (b)-(d) for up to 18 cycles, and (f) 72 °C for 180 s. The amplified biotinylated double-stranded DNAs (dsDNAs) were purified using Nucleospin Gel and a PCR Clean-up kit and then reacted with the Dynabeads™ Myone™ Streptavidin C1 magnetic beads in coupling buffer (5 mM Tris-HCl [pH 7.5], 1 M NaCl, 0.5 mM EDTA, and 0.0025% [v/v] Tween 20) for 1 h at 25 °C. After the bead-dsDNA complexes were washed using a coupling buffer and an external magnetic separator, the ssDNAs were eluted by adding 0.2 M NaOH and purified via ethanol precipitation process. The ssDNA library solution was used for the next SELEX round after measuring the concentration using a UV–Vis spectrophotometer.

In negative selection, the magnetic beads were used instead of periostin to remove non-specific interactions of ssDNAs, and the unbound ssDNA solution was used as a library for the round of SELEX to obtain the periostin-ssDNA complex. After the 19th round of SELEX, the eluted bound ssDNA library was amplified with forward and reverse primers (Table S1), cloned into the vector using a pENTR D-TOPO cloning kit, and sequenced (Cosmogenetech, Seoul, South Korea). The Mfold program (http://www.unafold.org/mfold/applications/dna-folding-form.php)^36^ was utilized to analyze expected secondary structures of the aptamers, wherein 4 °C of folding temperature, 100 mM of NaCl, and 5 mM MgCl_2_ conditions were applied.

Two aptamer candidates were sequence optimized based on their predicted secondary structures and the randomized sequence region, with the resultant structures being compared with their original aptamers.

### Determination of the binding affinity of the two aptamers and their truncated forms

To verify the binding affinity of the aptamers and their truncated forms, a magnetic bead-based fluorescence assay was conducted^37^. Various concentrations of each 5′ Cy3 attached aptamers (0–200 nM, Table S3) were heated in boiling water for 5 min and then cooled on ice for 30 min in the binding buffer. His-tag isolation and pulldown magnetic beads were incubated with 200 pmol of periostin for 1 h at 25 °C. Then, the bead complexes were washed twice with the binding buffer. The prepared 5′ Cy3-labeled aptamer (0–200 nM) was allowed to react with the bead–protein complex for 1 h at 25 °C. All unbound aptamers were washed away using the binding buffer. The protein–aptamer complexes were collected from the magnetic beads with a binding buffer containing 500 mM imidazole and an external magnetic separator. Fluorescence signals were detected using a microplate reader ^38^. All experiments were conducted in triplicate. The dissociation constants (*K*_*d*_)were analyzed according to the equation y = (B_max_ × x)/(*K*_*d*_ + x) by fitting to the one-site (specific binding) model of GraphPad Prism 5 software (GraphPad, USA), where B_max_ is the maximum specific binding, and x is the concentration of each aptamer^39^.

### Verification of the simultaneous binding property of the two aptamers: two direct ELONAs

To run the assay, twofold serial dilutions of periostin solution (from 50 nM) were prepared in PBS buffer (137 mM NaCl, 2.7 mM potassium chloride, 10 mM sodium phosphate, and 1.8 mM potassium phosphate, pH 7.4) and loaded onto a 96-well plate. The plate was incubated overnight at 4 °C. After washing five times with 200 μL of PBS containing 0.05% (w/v) Tween 20, the plate was blocked by adding 200 μL of PBS containing 2% (w/v) BSA (PBS-B). The plate was again washed with 200 μL of PBS-T five times and then allowed to react with 100 μL of a 1 μM unmodified PL5_trunc_ (direct ELONA 1) and PL2_trunc_ (direct ELONA 2) aptamer solution for 1 h at 25 °C. After five washes with binding buffer, the plate was reacted with 100 μL of a 0.2 μM biotinylated PL2_trunc_ (direct ELONA 1) or PL5_trunc_ (direct ELONA 2) aptamer solution for 1 h. The plate was washed five times with the binding buffer, followed by the addition of 100 μL of streptavidin-horseradish peroxidase (200-fold diluted with binding buffer) and incubation for 30 min. Then, the plate was again washed five times using the binding buffer, followed by the addition of 100 μL of a TMB solution and incubation for 30 min. Thereafter, 100 μL of 2 M H_2_SO_4_ was added to stop the reaction, and the absorbance was measured using a microplate reader. The linearity of the absorbance was analyzed by fitting it to the simple linear regression of GraphPad Prism 5 software. All processes were performed in triplicate.

### Verification of the simultaneous binding property of the two aptamers: sandwich ELONA

To detect periostin, 100 μL of 0.2 μM H_2_N-A_10_PL2_trunc_ aptamers were conjugated with a maleic anhydride-activated 96-well plate for 90 min at 25 °C. The plate was washed five times with PBS-T and then blocked with 200 μL of PBS-B for 1 h at 25 °C. The plate was again washed five times with PBS-T. Serial dilutions of periostin (from 50 nM) were prepared and reacted with the plate for 1 h at 25 °C. The plate was then washed five times with 200 μL of binding buffer. Next, 100 μL of 1 μM biotinylated PL5_trunc_ aptamers were prepared and reacted with the plate for 1 h at 25 °C. The plate was washed five times with 200 μL of binding buffer and then reacted with 100 μL of a 500-fold diluted streptavidin-poly HRP for 30 min at 25 °C. The plate was washed five times with 200 μL of binding buffer and then reacted with a TMB solution for 30 min at 25 °C. The reaction was stopped by adding 100 μL of 2 M H_2_SO_4_, and absorbance at λ = 450 nm was measured using a microplate reader. All experiments were performed in triplicate. The linearity of the absorbance was analyzed by fitting it to the simple linear regression of GraphPad Prism 5 software.

### Preparation of AuNPs, Au@RiTC NPs, Au nanoprobes, and Ag nanoprobes

The AuNPs (12 nm) were synthesized using the citrate reduction method ^40^. Briefly, 50 mL of a 1 mM gold (III) chloride solution was heated to reflux through vigorous stirring. The 5 mL of 38.8 mM trisodium citrate solution was rapidly injected into the boiling Au solution. When its color changed to burgundy red, the solution was cooled down slowly to 25 °C under vigorous stirring. The resultant AuNP solution was then stored at 4 °C until further use.

The Au@RiTC NPs were synthesized using a previously described method ^23^. RiTC dye solutions at various concentrations were added to a 1 nM AuNP solution and incubated overnight at 25 °C with orbital shaking (200 rpm). The synthesized Au@RiTC NPs solutions were then stored at 4 °C until further use.

Au nanoprobes were synthesized using the following method: 0.1 μM of the HS-A_10_PL2_trunc_ aptamer was allowed to react with a 1 μM TCEP-HCl solution for 30 min at 25 °C. The aptamer solution was then added to a 1 nM Au@RiTC NPs solution and incubated for 30 min at 25 °C with orbital shaking (200 rpm). The concentration of NaCl was increased to 0.8 M using the salt-aging method to ensure the attachment of the aptamers onto the surface of the AuNPs^30^. Synthesized Au nanoprobes were centrifuged for 40 min at 17 000 × *g*, the supernatant was removed, and 0.01% (v/v) sodium dodecyl sulfate (SDS) was added. This process was repeated three times, and the synthesized Au nanoprobe solution was stored at 4 °C until further use.

To fabricate the Ag nanoprobes, 0.4 μM of the HS-A_10_P5_trunc_ aptamer was allowed to react with a 4 μM TCEP solution for 30 min at 25 °C. The aptamer solution was then added to a 2 pM AgNP solution. After incubation for 30 min at 25 °C, the concentration of NaCl was increased to 0.8 M. The synthesized Ag nanoprobes were centrifugated for 30 min at 9 000 × *g*, the supernatant was removed, and 0.01% (v/v) SDS was added. This process was repeated three times, and the synthesized Ag nanoprobe solution was stored at 4  °C until further use.

### Detection of periostin using the metal-enhanced fluorescence nanosensor

To run this assay, two-fold serial dilutions of a periostin solution (from 50 nM stock solution) were prepared and mixed with the Ag and Au nanoprobes (at final concentrations of 0.02 pM and 0.2 nM, respectively) in a 96-well black plate. After the plate was incubated for 30 min at 25 °C with orbital shaking (200 rpm), fluorescence was measured using a microplate reader. Experiments were performed in triplicate. The linearity of the fluorescence was analyzed by fitting to the simple linear regression of GraphPad Prism 5 software (GraphPad, USA). The limit of detection (LOD) was calculated as (3 × σ)/S, where σ is the standard deviation (s.d.) of the sample fluorescence, and S is the slope of the calculated calibration curve. The coefficient of variation (CV) was calculated as (s.d./mean) × 100, where the s.d. and mean are the standard deviation and mean fluorescence measured at each periostin concentration, respectively. The recovery value was calculated as the percentage of the practical concentration divided by the theoretical concentration.

### Specificity test of the fluorescence nanosensor in buffer

For this assay, 10 nM of periostin and 100 nM of HSA, fibrinogen, and γ-globulin were prepared and allowed to react with the Au and Ag nanoprobes. The next process was the same as that described under *Detection of periostin using the metal-enhanced fluorescence nanosensor*. Measurements were taken thrice.

### Detection of periostin using the fluorescence nanosensor in spiked human serum

For this assay, 200 μL of a two-fold serially diluted periostin solution (from a 100 nM stock solution) containing 10% (v/v) of human serum was prepared and allowed to react with the Au and Ag nanoprobes. The remaining steps were the same as those described under *Detection of periostin using the metal-enhanced fluorescence nanosensor*. All measurements were taken in triplicate.

## Supplementary Information

Supplementary Information.

## Data Availability

The protein sequences used during the study are available in the UniProtKB, Q15063.
