# Supplementary material for: Development of a one-shot dual aptamer-based fluorescence nanosensor for rapid, sensitive, and label-free detection of periostin
Source: Sci Rep. 2023 Jun 23;13:10224. doi: 10.1038/s41598-023-37418-0 (PMC10290134; doi:10.1038/s41598-023-37418-0)
Supplement: Supplementary file 1 — Supplementary Information. [file 41598_2023_37418_MOESM1_ESM.pdf]

Supplementary Information (SI)

Development of a one-shot dual aptamer-based fluorescence nanosensor for rapid, sensitive, and label-free detection of periostin

Jonghoon Park and Changill Ban*

Department of Chemistry, Pohang University of Science and Technology, 77, Cheongam-Ro, Nam-Gu, Pohang, Gyeongbuk, 37673, Republic of Korea.

* Corresponding author

C. Ban

Department of Chemistry, Pohang University of Science and Technology, 77, Cheongam-Ro, Nam-Gu, Pohang, Gyeongbuk 37673, Republic of Korea

E-mail: ciban@postech.ac.kr; Tel: +82-54-279-2127; Fax: +82-54-279-8649

Table of Contents

1. Supplementary Tables and Figures

1.1. Tables S1–S7

1.2. Figures S1–S13

1. Supplementary Tables and Figures

1.1. Supplementary Tables S1-S7

Table S1. Sequence information of the single-stranded DNA (ssDNA) library and each primer used for in vitro selection

Name	Sequence (5′ → 3′)	Length (nt)
**ssDNA library**	CAC CTA ATA CGA CTC ACT ATA GCG GAT CCG A N_{40} CTG GCT CGA ACA AGC TTG C	90
**Forward primer**	CAC CTA ATA CGA CTC ACT ATA GCG GA	26
**Reverse primer**	GCA AGC TTG TTC GAG CCA G	19
**Reverse primer (5′ biotin)**	Biotin-GCA AGC TTG TTC GAG CCA G	19

The ssDNA library consisted of forward and reverse primers and 40 random nucleotides. Red text indicates the random sequence region. nt; nucleotide.

Table S2. Information about the two aptamers (PL2 and PL5) and their truncated forms

Name	Sequence (5′ → 3′)	Length (nt)
**PL2**	CAC CTA ATA CGA CTC ACT ATA GCG GA TCC GAA AGT CAA CGC CAG ATA AAA ACA ATT TGC CTT CCT CCG CCC CTG GCT CGA ACA AGC TTG C	90
**PL5**	CAC CTA ATA CGA CTC ACT ATA GCG GA TCC GAT TCG AACC AGA ATA TTA GTC GCA AAA TCT TGA TGC AGT CA CTG GCT CGA ACA AGC TTG C	90
**PL2trunc**	TCA ACG CCA GAT AAA AAC AAT TTG CCT TCC TCC GCC CCT GGC TCG AA	47
**PL5trunc**	TAA TAC GAC TCA CTA TAG CGG ATC CGA TTC GAA CCA GAA TAT TAG TCG CAA AAT CTT GAT G	61

Red text indicates the random sequence region. nt, nucleotide.

Table S3. Information about all the aptamers used in the magnetic bead-based fluorescence assay

Name	Modification (5′)	Sequence (5′ → 3′)	Length (nt)
**PL2**	Cy3	CAC CTA ATA CGA CTC ACT ATA GCG GA TCC GAA AGT CAA CGC CAG ATA AAA ACA ATT TGC CTT CCT CCG CCC CTG GCT CGA ACA AGC TTG C	90
**PL5**		CAC CTA ATA CGA CTC ACT ATA GCG GA TCC GAT TCG AACC AGA ATA TTA GTC GCA AAA TCT TGA TGC AGT CA CTG GCT CGA ACA AGC TTG C	90
**$PL2_{trunc}$**		TCA ACG CCA GAT AAA AAC AAT TTG CCT TCC TCC GCC CCT GGC TCG AA	47
**$PL5_{trunc}$**		TAA TAC GAC TCA CTA TAG CGG ATC CGA TTC GAA CCA GAA TAT TAG TCG CAA AAT CTT GAT G	61

All functional groups were conjugated to the 5′ regions of each aptamer. Red text indicates the random sequence region. nt, nucleotide.

Table S4. Sequence information on all aptamers used in direct and sandwich ELONAs

Experiment	Aptamer	Modification (5′)	Sequence (5′ → 3′)	Length (nt)	Usage
**Direct ELONA 1**	$PL5_{trunc}$	-	TAA TAC GAC TCA CTA TAG CGG ATC CGA TTC GAA CCA GAA TAT TAG TCG CAA AAT CTT GAT G	61	Pre-treatment
	Biotin-$A_{10}PL2_{trunc}$	Biotin	A_{10} TCA ACG CCA GAT AAA AAC AAT TTG CCT TCC TCC GCC CCT GGC TCG AA	57	Detection
**Direct ELONA 2**	$PL2_{trunc}$	-	TCA ACG CCA GAT AAA AAC AAT TTG CCT TCC TCC GCC CCT GGC TCG AA	47	Pre-treatment
	Biotin-$A_{10}PL5_{trunc}$	Biotin	A_{10} TAA TAC GAC TCA CTA TAG CGG ATC CGA TTC GAA CCA GAA TAT TAG TCG CAA AAT CTT GAT G	71	Detection
**Sandwich ELONA**	Biotin-$A_{10}PL5_{trunc}$	Amine (-NH_2)	A_{10} TCA ACG CCA GAT AAA AAC AAT TTG CCT TCC TCC GCC CCT GGC TCG AA	57	Capture
	H_2N-$A_{10}PL2_{trunc}$	Biotin	A_{10} TAA TAC GAC TCA CTA TAG CGG ATC CGA TTC GAA CCA GAA TAT TAG TCG CAA AAT CTT GAT G	71	Detection

All functional groups were attached to the 5′ regions of each aptamer.

Table S5. Sequence information on the modified aptamers used in the synthesis of Au and Ag nanoprobes

Name	Modification (5′)	Sequence (5′ → 3′)	Length (nt)	Applied NPs
**HS-A_{10}PL2$_{trunc}$**	Thiol (HS)	A_{10} TCA ACG CCA GAT AAA AAC AAT TTG CCT TCC TCC GCC CCT GGC TCG AA	57	Au@RiTC NPs
**HS-A_{10}PL5$_{trunc}$**	Thiol (HS)	A_{10} TAA TAC GAC TCA CTA TAG CGG ATC CGA TTC GAA CCA GAA TAT TAG TCG CAA AAT CTT GAT G	71	AgNPs

All thiol groups were conjugated to the 5′ regions of each aptamer. NP, nanoparticle

Table S6. Coefficients of variation (a) and recovery percentages (b) of the fluorescence nanosensor in buffer.

a)

Periostin (nM)	Mean of fluorescence intensity	s.d. of fluorescence intensity	Coefficient of variation (%)
**0.78**	2071.00	34.22	1.65
**1.56**	2647.00	52.85	2.00
**3.13**	4167.00	38.22	0.92
**6.25**	7299.67	107.68	1.48

b)

Periostin (nM)	Theoretical concentration (mean ± s.d.)	Recovery (%)
**0.78**	0.89 ± 0.04	114.10
**1.56**	1.48 ± 0.05	94.87
**3.13**	3.05 ± 0.04	97.44
**6.25**	6.29 ± 0.11	100.64

Table S7. Coefficients of variation (a) and recovery percentages (b) of the fluorescence nanosensor in 10% (v/v) spiked human serum

a)

Periostin (nM)	Mean of fluorescence intensity	s.d. of fluorescence intensity	Coefficient of variation (%)
**0.78**	2525.33	63.52	2.52
**1.56**	3165.00	71.39	2.26
**3.13**	4755.67	74.65	1.57
**6.25**	7004.67	36.07	0.51

b)

Periostin (nM)	Theoretical concentration (mean ± s.d.)	Recovery (%)
**1.56**	1.39 ± 0.15	89.10
**3.13**	2.95 ± 0.17	94.25
**6.25**	6.81 ± 0.18	108.96
**12.50**	12.28 ± 0.09	98.24

2.2. Supplementary Figures S1-S10

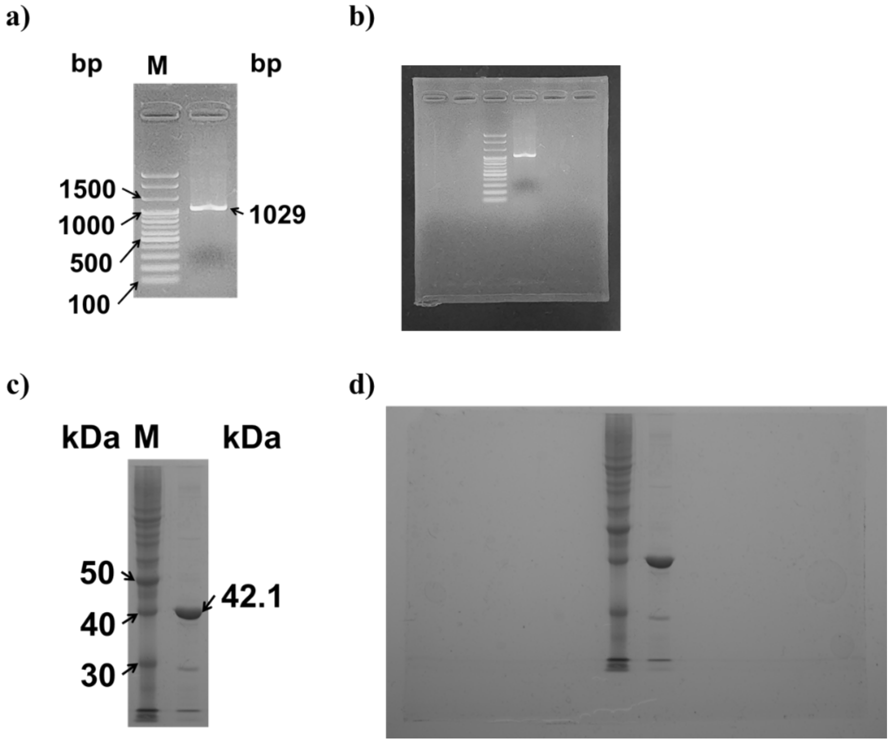

Figure S1. Gel electrophoresis of the recombinant periostin gene and protein.

a) The PCR product (1029 bp) of the periostin gene on a 3% (w/v) agarose gel in 0.5× TBE buffer, and b) the original image of (a). The BioFACT^{TM} 100 bp plus DNA ladder was used as a marker. c) The purified periostin protein (42.1 kDa) on a 12.5 % (v/v) SDS-PAGE gel, and d) the original image of (c). Step-view 10 kDa marker (right) was used as a marker. The images in a) and c) have been cropped from b) and d), respectively and enlarged for readability. M: marker, bp: base pair, kDa: kilodalton.

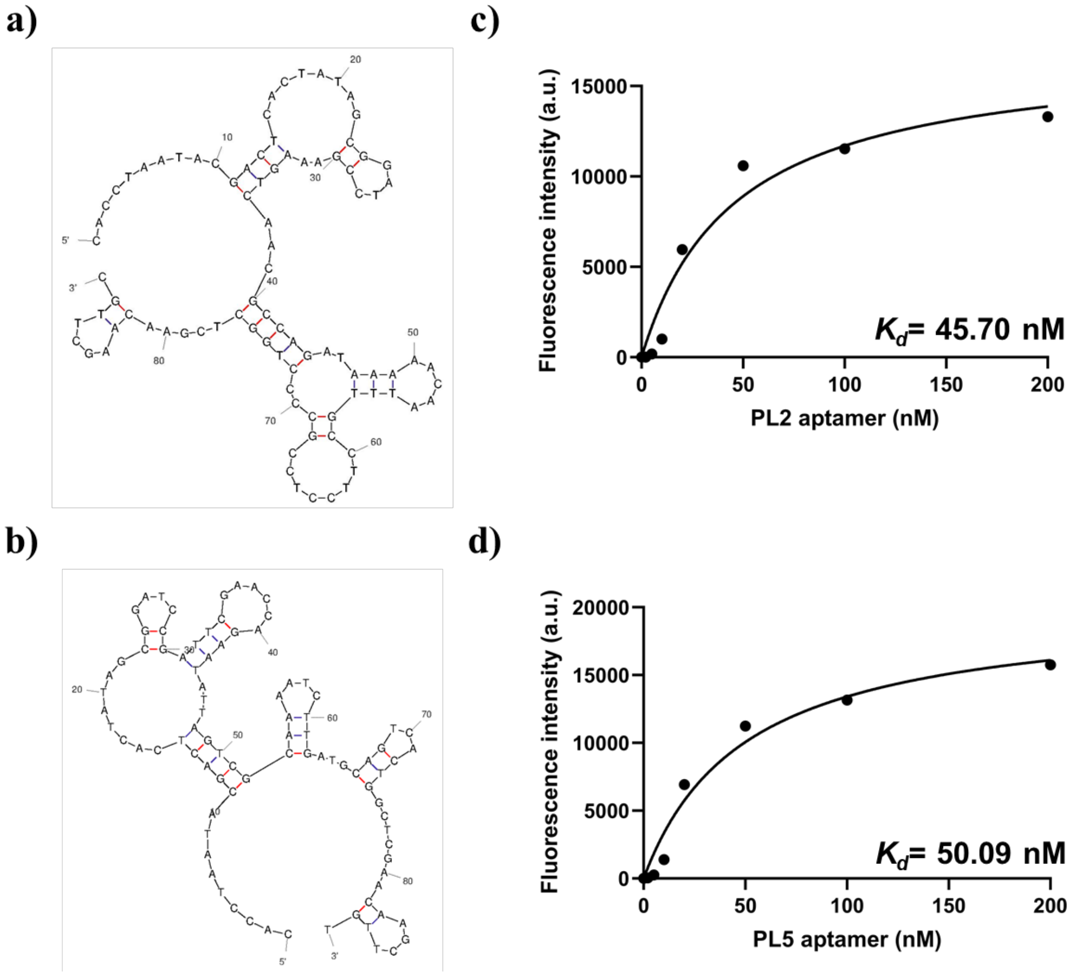

Figure S2. Predicted secondary structure and binding affinity of PL2 and PL5 aptamers

Predicted secondary structures of the (a) PL2 and (b) PL5 aptamers using the Mfold program (http://www.unafold.org/mfold/applications/dna-folding-form.php). Binding curves of the (c) PL2 and (d) PL5 aptamers. The black line represents the saturation curve of aptamers for periostin. The calculated K_d values of the PL2 and PL5 aptamers were 45.70 and 50.09 nM, respectively. n = 3.

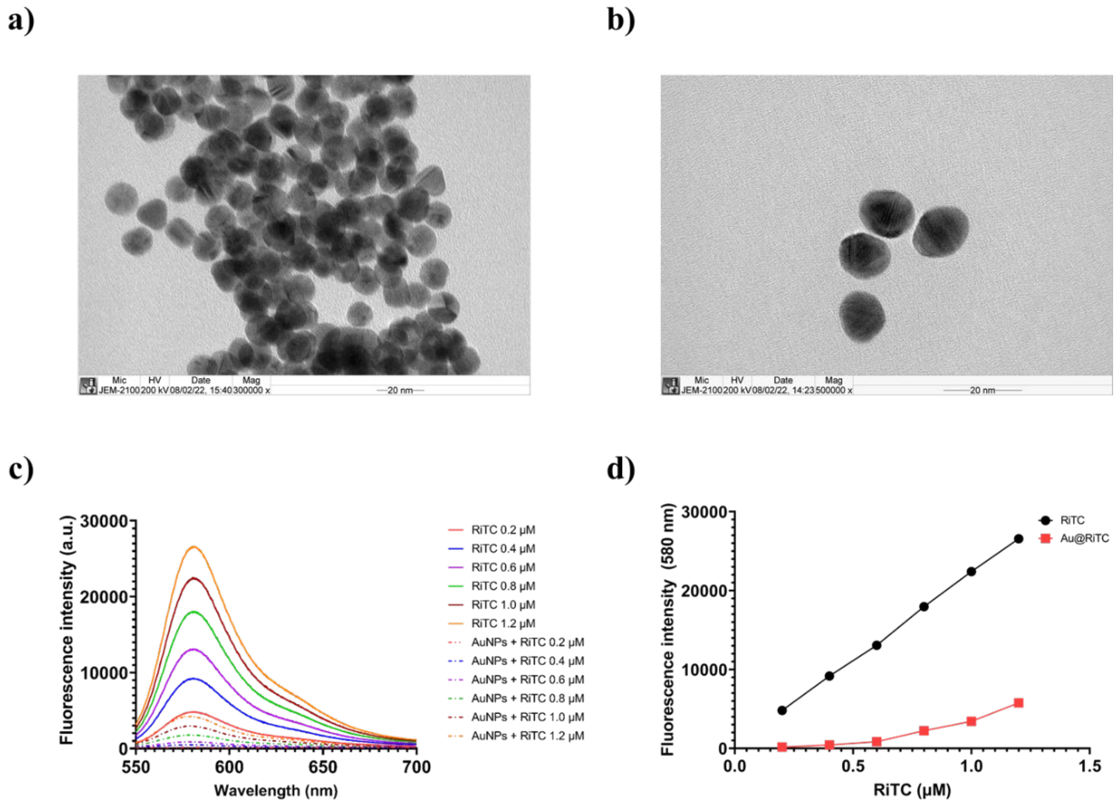

Figure S3. Physical characteristics of the synthesized Au@RiTC NPs.

TEM images of (a) AuNPs and (b) Au@RiTC NPs. Both NPs showed a size of 12 nm. (c) Fluorescence spectra of various concentrations of free RiTC solution and Au@RiTC NPs solution. AuNPs significantly quenched the fluorescence of RiTC. (d) Fluorescence spectra of free RiTC solution and Au@RiTC NPs solution at 580 nm. The free RiTC solution exhibited linear fluorescence intensity with the concentration of RiTC, but the fluorescence intensity of Au@RiTC slightly increased after treatment with RiTC above 0.8 μM.

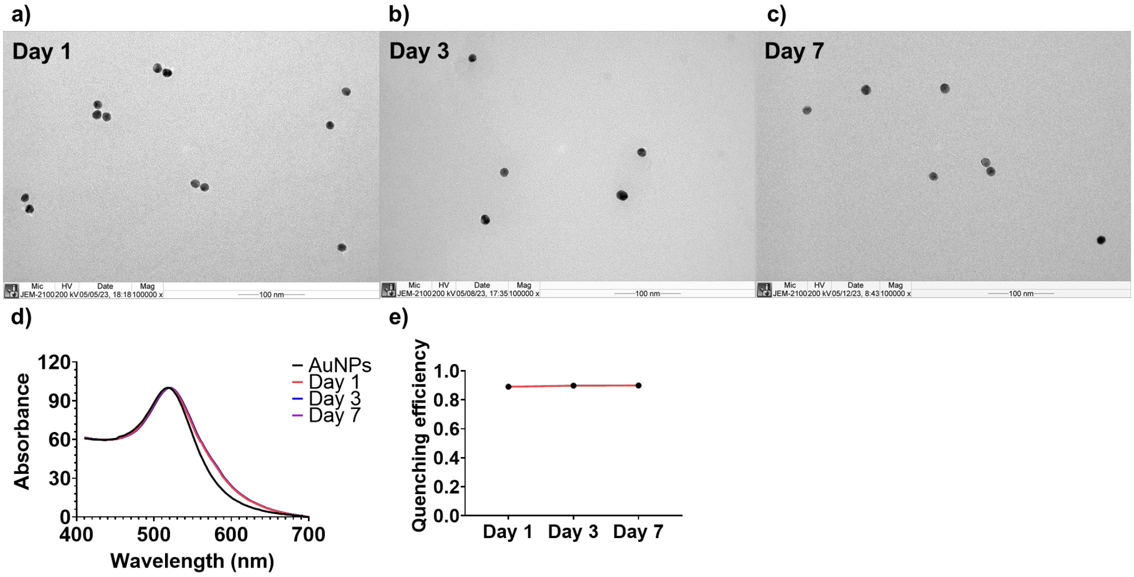

Figure S4. Stability test of the synthesized Au@RiTC NPs.

TEM images of Au@RiTC NPs measured (a) one day, (b) three days, and (c) seven days after synthesis. The synthesized Au@RiTC NPs showed stable dispersion for one week. The core size of NPs was 12 nm. d) Normalized UV-Vis spectra of bare AuNPs and Au@RiTC NPs measured on days 1, 3, and 7 after synthesis. The synthesized Au@RiTC NPs showed absorption spectra similar to those of AuNPs regardless of the timepoint since synthesis. (e) Change in quenching efficiency of Au@RiTC NPs with time after synthesis. The synthesized Au@RiTC NPs showed ~ 90% quenching efficiency, which was maintained for one week.

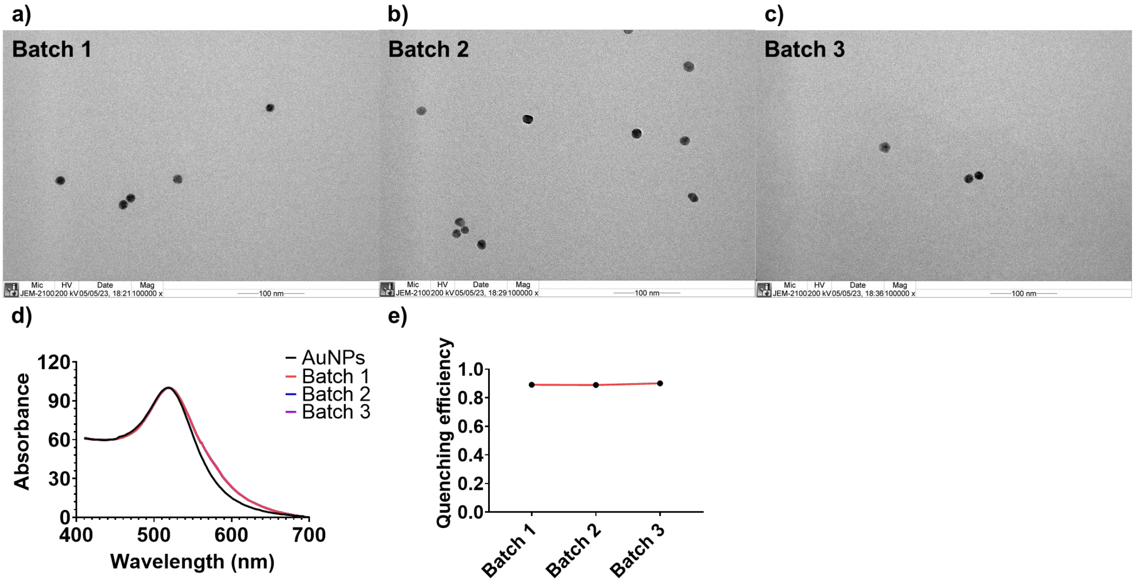

Figure S5. Reproducibility test of the synthesized Au@RiTC NPs.

TEM images of each Au@RiTC NPs synthesized in (a) batch 1, (b) batch 2, and (c) batch 3. The synthesized Au@RiTC NPs showed stable dispersion in all batches, and the core size of the NPs was 12 nm. d) Normalized UV-Vis spectra of bare AuNPs and Au@RiTC NPs synthesized in different batches. The synthesized Au@RiTC NPs showed similar absorption spectra regardless of the batch. (e) Comparison of quenching efficiency of Au@RiTC NPs synthesized in different batches. The synthesized Au@RiTC NPs showed ~90% quenching efficiency in all batches.

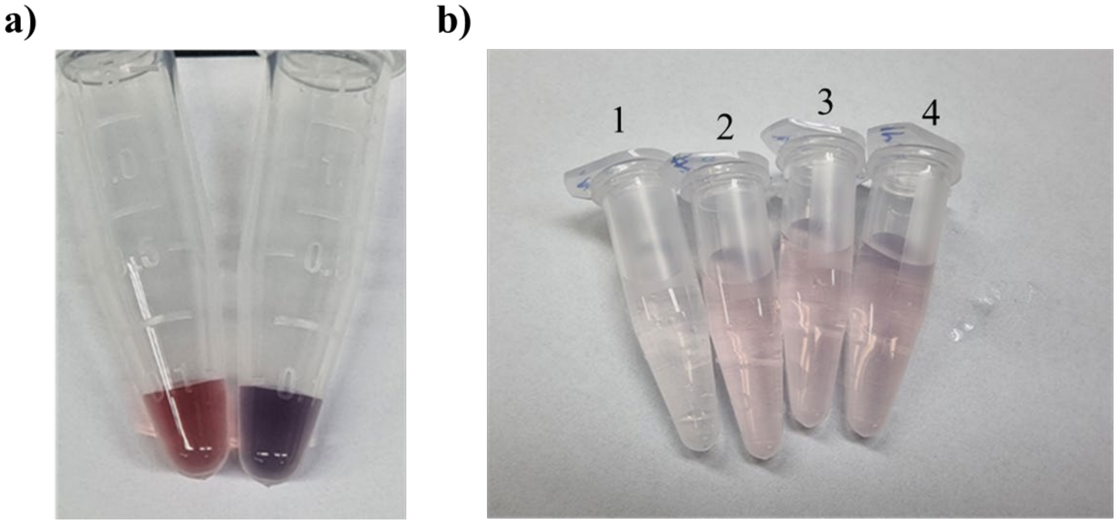

Figure S6 Confirmation of the color change in solutions of Au@RiTC NPs and Au nanoprobes after synthesis and purification.

(a) Image of the Au@RiTC NP solution before (left) and after (right) a high-speed centrifugation process. The color of the solution changed from red to darl violet after the centrifugation process. (b) Image of each synthesized Au nanoprobe after the purification process. The solutions in tubes 1–4 correspond to the reaction of Au@RiTC with 50, 100, 150, and 200 nM of the thiolated $PL2_{trunc}$ aptamer, respectively. The solution color of all synthesized Au nanoprobes showed a distinct red color except for tube 1.

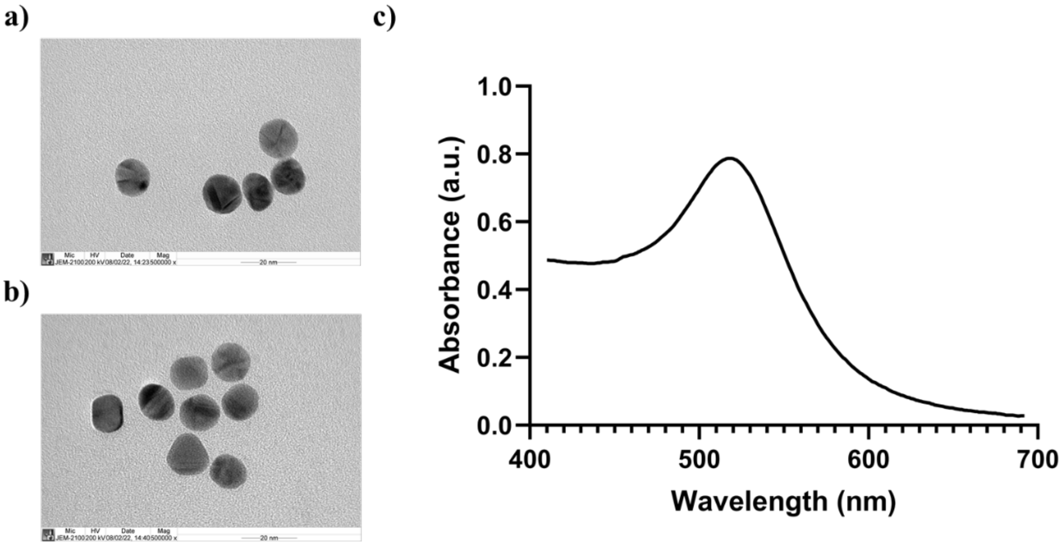

Figure S7. Physical characteristics of AuNPs and the synthesized Au nanoprobes.

TEM images of (a) Au@RiTC NPs and (b) Au nanoprobes. Both NPs had a size of 12 nm. (c) The UV-Vis spectrum of the 12 nm AuNPs.

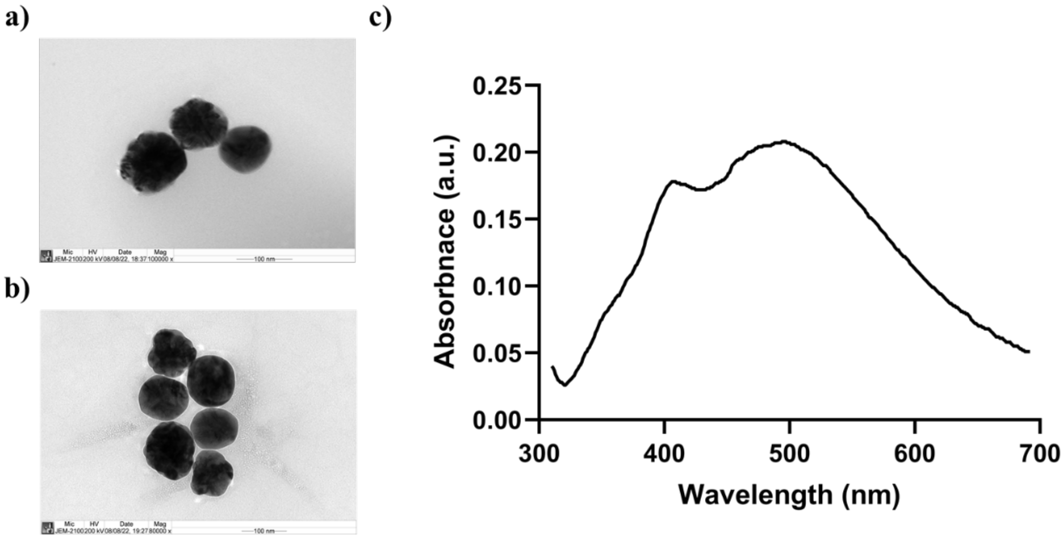

Figure S8. Verification of physical properties of AgNPs and Ag nanoprobes.

TEM images of (a) AgNPs and (b) synthesized Ag nanoprobes; both had a size of 100 nm. (c) The UV-Vis spectrum of 100 nm AgNPs.

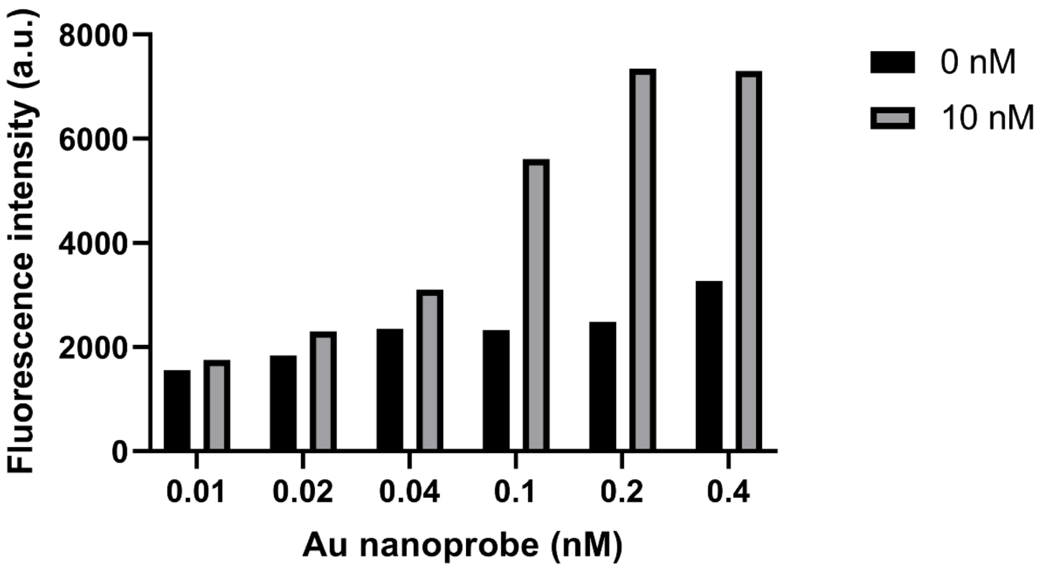

Figure S9. Fluorescence intensity of the nanosensor when various concentrations (500, 1,000, 2,000, 5,000, 10,000, and 20,000 times) of Au nanoprobes were reacted with periostin at 0.02 pM Ag nanoprobe concentration.

Significant fluorescence intensity of the nanosensor began to appear when treated with 0.1 nM Au nanoprobe and reached a maximum when treated with 0.2 nM Au nanoprobe. The fluorescence intensity of the blank increased when treated with 0.4 nM Au nanoprobes. No continuous fluorescence regeneration (regardless of the presence or absence of periostin) or quenching (in the presence of periostin) was observed in the nanosensor.

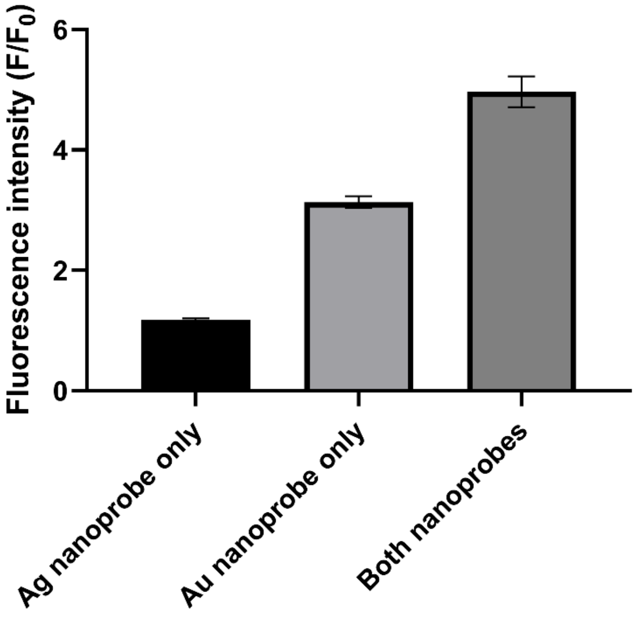

Figure S10. Verification of the MEF effect of Ag nanoprobes in the regeneration of the quenched fluorescence of the nanosensor.

This experiment was assessed by treating periostin respectively with Ag nanoprobes only, Au nanoprobes only, or both nanoprobes. Although treatment with Au nanoprobe only showed a meaningful fluorescence intensity, the treatment with both nanoprobes showed further increased fluorescence intensity. Bars: ± s.d., n = 3.

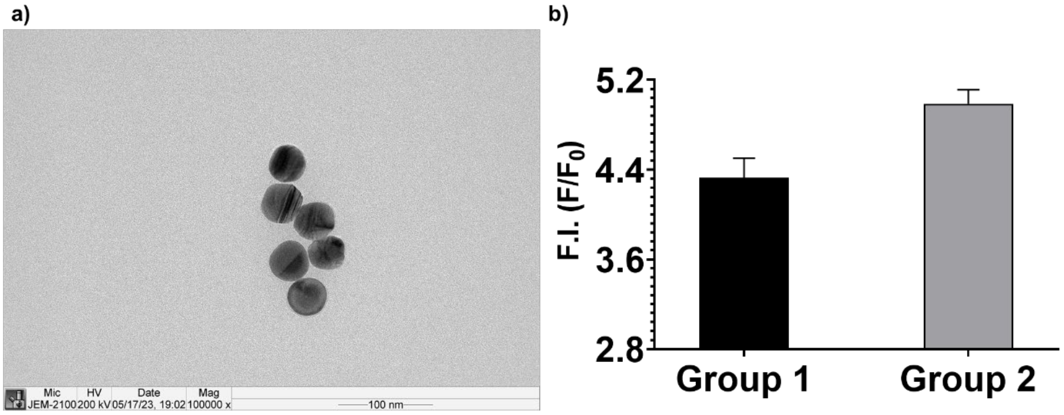

Figure S11. Verification of the effect of AgNPs of different sizes on periostin detection.

a) TEM image of 40 nm Ag nanoprobes. b) Fluorescence intensity of each nanosensor. In Group 1, the Ag nanoprobes synthesized with 40 nm AgNPs were applied to detect periostin. Group 2 used the Ag nanoprobes synthesized with 100 nm AgNPs for detecting periostin. Both groups exhibited significant fluorescence intensities, with group 2 showing stronger fluorescence intensity. Fluorescence intensity was measured by applying 0 nM and 10 nM periostin solutions to both groups. Bars: ± s.d., n = 3.

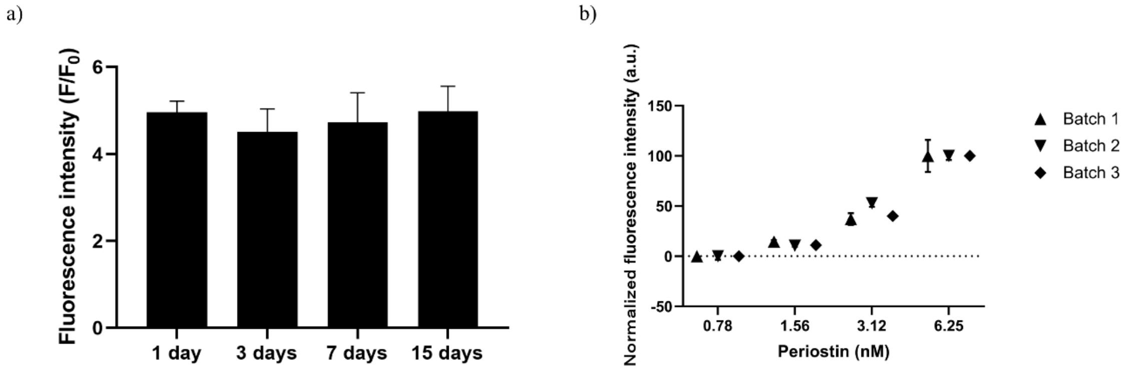

Figure. S12. Verification of the stability and reproducibility of the nanosensor.

(a) Detection performance tests of the nanosensor measured at 1, 3, 7, and 15 days after synthesis of the two nanoprobes. The nanosensor showed constant fluorescence intensity regardless of the timepoint. (b) Reproducibility test of the fluorescence nanosensor for periostin detection in dynamic range (0.78–6.25 nM). The nanosensor showed similar detection performance for each protein concentration in all batches. Each batch represents periostin detection in an independent condition. Bars: ± s.d., n = 3.

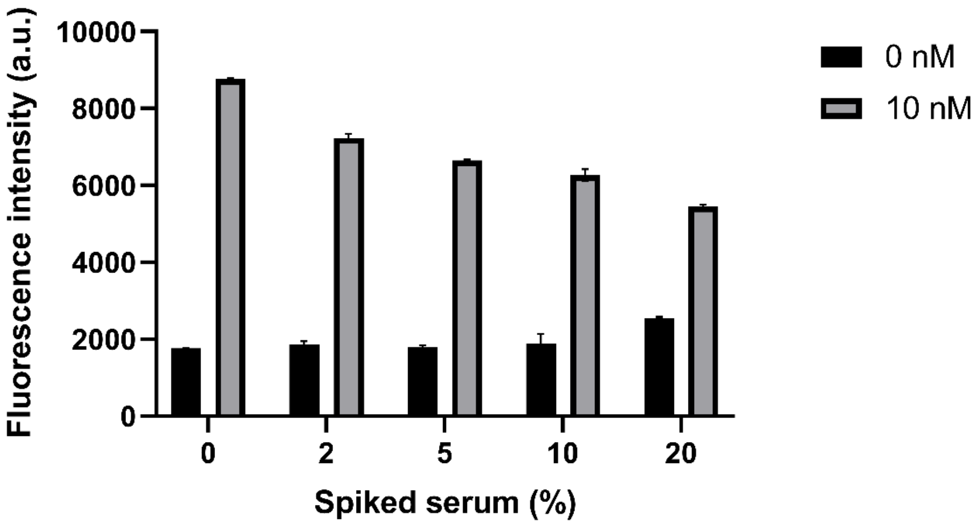

Figure S13. Fluorescence intensity of the nanosensor in various dilutions of human serum spiked with periostin.

The fluorescence intensity of periostin decreased as the dilution ratio of human serum was increased, but decreased significantly under 20% diluted human serum. The blank signal of the nanosensor was maintained up to 10% dilution of human serum but markedly increased under 20% diluted human serum. Bars: ± s.d., n = 3.
